# Determinants of improvement trends in health workers’ compliance with outpatient malaria case-management guidelines at health facilities with available “test and treat” commodities in Kenya

**DOI:** 10.1371/journal.pone.0259020

**Published:** 2021-11-05

**Authors:** Beatrice Amboko, Kasia Stepniewska, Lucas Malla, Beatrice Machini, Philip Bejon, Robert W. Snow, Dejan Zurovac

**Affiliations:** 1 KEMRI-Wellcome Trust Research Programme, Nairobi, Kenya; 2 WorldWide Antimalarial Resistance Network, Oxford, United Kingdom; 3 Centre for Tropical Medicine and Global Health, University of Oxford, Oxford, United Kingdom; 4 Division of National Malaria Programme, Ministry of Health, Nairobi, Kenya; PLOS: Public Library of Science, UNITED KINGDOM

## Abstract

**Background:**

Health workers’ compliance with outpatient malaria case-management guidelines has been improving in Africa. This study examined the factors associated with the improvements.

**Methods:**

Data from 11 national, cross-sectional health facility surveys undertaken from 2010–2016 were analysed. Association between 31 determinants and improvement trends in five outpatient compliance outcomes were examined using interactions between each determinant and time in multilevel logistic regression models and reported as an adjusted odds ratio of annual trends (T-aOR).

**Results:**

Among 9,173 febrile patients seen at 1,208 health facilities and by 1,538 health workers, a higher annual improvement trend in composite “test and treat” performance was associated with malaria endemicity-lake endemic (T-aOR = 1.67 annually; p<0.001) and highland epidemic (T-aOR = 1.35; p<0.001) zones compared to low-risk zone; with facilities stocking rapid diagnostic tests only (T-aOR = 1.49; p<0.001) compared to microscopy only services; with faith-based/non-governmental facilities compared to government-owned (T-aOR = 1.15; p = 0.036); with a daily caseload of >25 febrile patients (T-aOR = 1.46; p = 0.003); and with under-five children compared to older patients (T-aOR = 1.07; p = 0.013). Other factors associated with the improvement trends in the “test and treat” policy components and artemether-lumefantrine administration at the facility included the absence of previous RDT stock-outs, community health workers dispensing drugs, access to malaria case-management and Integrated Management of Childhood Illness (IMCI) guidelines, health workers’ gender, correct health workers’ knowledge about the targeted malaria treatment policy, and patients’ main complaint of fever. The odds of compliance at the baseline were variable for some of the factors.

**Conclusions:**

Targeting of low malaria risk areas, low caseload facilities, male and government health workers, continuous availability of RDTs, improving health workers’ knowledge about the policy considering age and fever, and dissemination of guidelines might improve compliance with malaria guidelines. For prompt treatment and administration of the first artemether-lumefantrine dose at the facility, task-shifting duties to community health workers can be considered.

## Introduction

Malaria continues to be a major public health problem in Africa and case-management is a key component to reducing the malaria burden [1, 2]. The global shift from presumptive treatment of fevers to the 2010 “test and treat” policy recommending parasitological testing of all suspected malaria cases and targeted antimalarial treatment for only confirmed cases presented a major milestone in the history of malaria case-management [3, 4].

Health workers’ compliance with guidelines is one of the key aspects determining the cost-effectiveness of the “test and treat” policy implementation [5–7]. Numerous outpatient malaria case-management studies across Africa have shown that health workers’ clinical practices are often characterised by non-compliance with testing recommendations [8, 9], use of non-recommended antimalarials for confirmed cases [10, 11], irrational antimalarial treatments for test-negative patients [10, 12, 13] and missed opportunities for the administration of prompt antimalarial treatment at health facilities [8, 14–16].

Moreover, non-compliant practices have not only been observed in clinical settings where lack of “test and treat” commodities for malaria preclude compliance with guidelines [17], but also at facilities with adequate availability of malaria diagnostic and treatment commodities [14, 15, 18]. Besides the commodities, a variety of determinants may influence health workers’ compliance with guidelines [19–21]. According to Rowe et al. [19], these determinants can be grouped into two categories, interventional (e.g. training, guidelines, supervision) and non-interventional (e.g. patients’ age, gender, the severity of illness). The strongest study design for evaluating interventions to improve health workers’ compliance is randomized controlled trials as they can show causality, while observational studies can only establish associations. However, when a policy is implemented on a large scale under real-life conditions, the use of observational studies can be suitable and is often the only feasible option to identify both interventional and non-interventional determinants of performance [19]. Recent studies across Africa have suggested a variety of factors associated with health workers’ compliance with the test-based management of malaria [8, 22–24]. However, these studies were commonly undertaken at a single point in time [8, 22], focusing on only one of the outcomes (e.g. only testing) [23], and none examined determinants of the improvements in compliance with guidelines over time to assess the factors associated with long-term change in practices.

Improvements in the “test and treat” compliance have been observed on various scales across Africa [8, 12, 15, 25]. Such improvements have been well described in Kenya, where between 2010 and 2016, health workers’ compliance with all key outpatient case-management indicators significantly increased [23, 26, 27]. The differences in compliance trends across malaria epidemiological zones in Kenya have been previously reported [28]. In this paper, the effects of 31 interventional and non-interventional determinants that might be associated with the improvement trends in health workers’ compliance with malaria case-management guidelines at health facilities with available diagnostic and treatment commodities for malaria were examined.

## Methods

### Outpatient malaria case-management standards and implementation context

Kenya adopted the “test and treat” policy recommending universal parasitological testing of all patients with fever across all areas of malaria transmission with either malaria microscopy or rapid diagnostic tests (RDTs), and subsequent antimalarial treatment for only test positive patients in 2010 [29]. The recommended first-line treatment for uncomplicated malaria has been artemether-lumefantrine (AL) since 2006 [30, 31]. For prompt treatment and promotion of patients’ adherence to medicines, administration of the first AL dose is recommended at the health facility at the time when drugs are dispensed.

**Fig 1:**
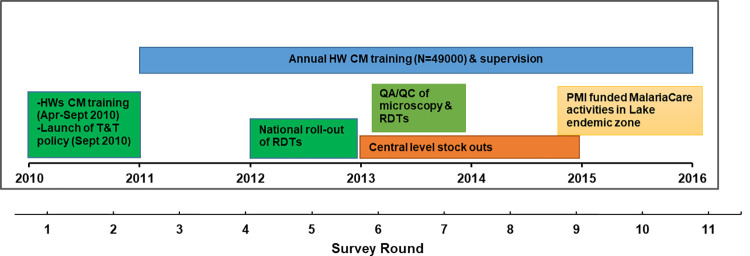
Timeline of key implementation activities of malaria diagnosis and treatment policy in Kenya. ^*^HWs-health workers; CM-Case-management; T&T- “test and treat”; PMI-President’s malaria Initiative; QA/QC- quality assurance/quality control; RDTs- rapid diagnostic tests.

To support the countrywide translation of the 2010 malaria case-management policy into clinical practice, a series of routine programmatic activities have been implemented (Fig 1). Between April and September 2010, the first nationwide in-service training of frontline health workers on the new case-management policy was undertaken followed by the launch of the new guidelines in September 2010 [23, 29]. Subsequently, nationwide rounds of 3-day in-service malaria case-management training have been undertaken annually. The new malaria guidelines and accompanying job aids were distributed through routine commodity supply channels and during the in-service training. Moreover, in 2012, there was a national scale-up of RDTs to support the parasitological diagnosis of malaria and promote the appropriate use of antimalarial drugs. Lastly, strengthening of malaria-specific supervision using a structured checklist to assess health workers’ capacity and training in malaria case-management status and practices, followed by feedback and on-the-job training has been rolled out nationally since 2011.

Other contextual factors during this period include; first, central level stock-outs of AL and RDTs due to a fire at the Kenya Medical Supplies Agency (KEMSA) stores in 2013 and levy tax that delayed the delivery of commodities for ten months in 2014. Second, the pilot implementation of laboratory quality assurance and quality control for malaria microscopy and RDTs in low transmission counties that included on-job training and mentorship from June to December 2013 [32, 33]. Finally, in 2015, MalariaCare, a USAID partnership, began working in the lake endemic zone to improve the quality of malaria diagnosis using microscopy and RDTs, and clinical case-management of malaria and other febrile illnesses [34]. The implemented activities included case-management and laboratory training, on-site supervision, and mentoring (known as “outreach training and support supervision, OTSS”), electronic data collection and follow up evaluation, and implementation of the lessons learned. By 2016, two rounds of OTSS were conducted, reaching 98% coverage of the facilities in the region, and this might have contributed to the high compliance levels noted in the zone (Fig 1) [35, 36].

### Data sources

**Fig 2:**
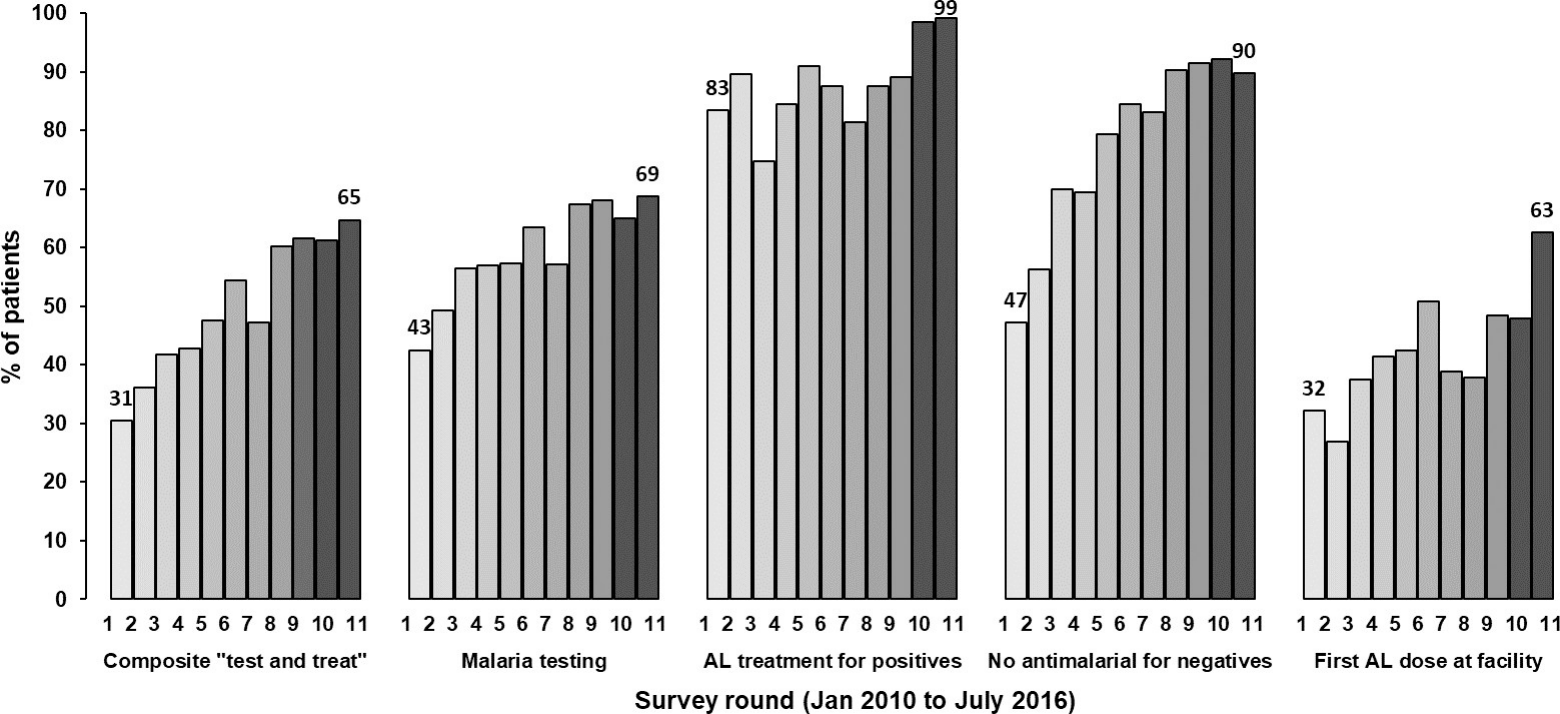
2010–2016 national trends in compliance with outpatient malaria “test and treat” and AL dispensing guidelines. AM- Antimalarial; AL-artemether-lumefantrine: the figure is based on health facilities with AL and malaria diagnostics available on the survey day.

The secondary analysis in this study utilised data from 11 national biannual cross-sectional, cluster sample health facility surveys undertaken between January 2010 and July 2016 (Fig 2). The primary monitoring indicator is a composite “test and treat” performance, measured at the patient level and comprised of malaria testing of febrile patients, AL treatment for test positive patients or no antimalarial for test negative patients. The sample size calculation details have been previously published [23, 26]. The sample size for each survey was calculated adjusting for the effect of clustering at the health facility level and the likelihood of practices at facilities without case-management commodities. For each survey, a proportionate stratified random sample of facilities was drawn from the Ministry of Health (MoH) master list of approximately 5,000 public health facilities taking into consideration the facility type, ownership and administrative boundaries to ensure national representativeness [37].

The number of assessed facilities ranged between 169 and 176 facilities per survey round. At each of the surveyed facilities, data collection methods included health facility assessments, interviews with health workers, and exit interviews with all eligible outpatients during one survey day when they were ready to leave the facility [23, 26, 28]. The patients’ exit interviews included all non-referred and non-pregnant patients weighing >5 kgs across all age groups and presenting for an initial visit with fever or history of fever. Information was collected from patient-held cards about malaria tests requested, test results reported, treatment prescribed, and direct questioning about patients’ demographics, presenting symptoms, prior use of antimalarials, and drug dispensing and counselling practices during the facility visit. Each facility was assessed to determine the availability of medicines and diagnostics services (RDTs or microscopy). Additionally, febrile patients’ caseload on the survey day, ownership, retrospective stock-outs of malaria commodities, the health worker cadre dispensing medicines, and the availability of support tools like malaria treatment guidelines and job aids were also assessed. Finally, all health workers who provided clinical consultations in the outpatient departments were interviewed. Information on their demographic characteristics, outpatient responsibility, pre-service training, access to guidelines and job aids, in-service training, supervision, knowledge about malaria “test and treat” policy and their perceptions of malaria endemicity was collected. Health workers’ knowledge about the malaria “test and treat” policy was assessed using self-administered true or false statements reflecting the national recommendations about fever as malaria testing criteria and antimalarial treatment for only test positive patients as the targeted treatment recommendation. Data quality was assured through five days of training of the field workers, double-entry into a Microsoft Access database, and comparisons of data files using a verification program in Microsoft Access and referring to paper-based questionnaires (S1-S3 Appendices).

### Outcomes and determinants

Five outcomes showing improvements in health workers’ compliance with national malaria case-management guidelines between 2010 and 2016 were selected (Fig 2). The outcomes reflected health workers’ decisions to test febrile patients for malaria, comply respectively with test positive and test negative results, deliver composite “test and treat” performance defined as a patient tested for malaria and prescribed AL if the test was positive or not prescribed an antimalarial if the test was negative, and provide prompt treatment by administering the first AL dose at the facility.

With respect to determinants, Box 1 outlines malaria endemicity, ten health facility-, 14 health worker- and six patient-level determinants examined for the association with the 2010–2016 improvements in health workers’ compliance with each of the five outcomes. The malaria endemicity classifications have been previously described [28]. In summary, the classifications included: 1) Lake endemic–high transmission areas around Lake Victoria in western Kenya with stable malaria transmission all year round; 2) Coast endemic—low to moderate transmission areas along the Indian Ocean coast; 3) Highland epidemic–areas of the western highlands with unstable, year-to-year variation in transmission; 4) Semi-arid, seasonal transmission–arid and semi-arid areas of northern, eastern and south-eastern Kenya with acute seasonal and low transmission; and 5) Low risk—areas of central highlands including Nairobi with low transmission. The determinants were selected a priori based on the conceptual framework developed by Rowe et al. [19] and included factors previously reported in the literature and some additional ones (like health worker cadre dispensing medicines, health workers’ perceptions of malaria endemicity, case complexity and prior use of antimalarials) hypothesised to affect compliance.

**Box 1. List of examined determinants**

**A) Malaria endemicity**
  1. Epidemiological zone (Lake endemic vs coast endemic vs highland epidemic vs semi-arid seasonal vs low)
**B) Health facility level**
  1. Facility ownership (government vs faith-based/non-governmental organisation (FBO/NGO))
  2. Level of outpatient care (dispensary vs health centre vs hospital)
  3. Febrile patients’ caseload on the survey day (≤25 vs >25 patients)
  4. Type of diagnostic test available at the facility (RDTs vs microscopy vs both)
  5. Stock-outs of 7 or more continuous days of RDTs in the past 3 months (Yes vs No)
  6. Stock-outs of 7 or more continuous days of AL in the past 3 months (Yes vs No)
  7. Absence of malaria microscopy in the past 3 months (Yes vs No)
  8. Availability of malaria guidelines (Yes vs No)
  9. Displayed malaria case-management chart (Yes vs No)
  10. Cadre dispensing drugs (CHW vs nurse/clinician vs pharmacist vs others)
**C) Health worker level**
  1. Health workers’ age (continuous)
  2. Gender (male vs female)
  3. Outpatient responsibility (in-charge vs others)
  4. Perception of malaria endemicity (high vs low)
  5. Cadre (nurse vs clinical/medical officer vs others)
  6. Malaria case-management in-service training (Yes vs No)
  7. Access to current malaria case-management guidelines (Yes vs No)
  8. Access to IMCI guidelines (Yes vs No)
  9. Any supervisory visit received in past 3 months (Yes vs No)
  10. Supervisory visit including any malaria case-management activity in the past 3 months (Yes vs No)
  11. Supervisory visit with the observation of consultation in the past 3 months (Yes vs No)
  12. Supervisory visit with feedback in the past 3 months (Yes vs No)
  13. Correct knowledge of malaria testing policy (Yes vs No)
  14. Correct knowledge of antimalarial treatment policy (Yes vs No)
**D) Patient-level**
  1. Age (<5 years vs >5 years), (0–11 months, 12–59 months, 5–14 years, 15–45 years, >45 years) and as a continuous variable
  2. Duration of illness (number of days)
  3. Temperature (continuous and <37.5 vs ≥ 37.5° C)
  4. Prior use of antimalarials for the same illness (Yes vs No)
  5. Main complaints (fever, cough, diarrhoea, headache, running nose, rash, vomiting, and chills)
  6. Case complexity (fever and other complaints vs fever only vs no fever)

### Statistical analysis

Since the absence of commodities precludes compliance with guidelines, the analysis was restricted to patients who visited facilities with malaria diagnostic services and AL available on survey days. Across all surveys, 517 (30%) health facilities, 598 (28%) health workers and 4,281 (31.8%) febrile patient consultations were excluded from the analysis after the restriction. Patient-level logistic regression models with random intercepts at the health facility level to adjust for clustering were used to assess the determinants of improvements in compliance. For each outcome, the probability *μ_ij_* that the patient *i* is correctly managed from a health facility *j* was modelled, and hence [*μ_ij_*/ (1-*μ_ij_*)] define the odds of health workers’ compliance. The annual trends in compliance with each binary outcome were first estimated using unadjusted multilevel logistic regression models with time in years as the only independent variable in the model (baseline model) and summarised as an odds ratio (OR) that represents the annual increase in the odds of compliance over time. The baseline model for the health workers’ compliance with each outcome was specified as:

$$logit\;(\mu_{ij})=\alpha +\beta t_{j}+\varepsilon_{ij}+\mu_{j}$$

where *α* is the intercept; *t* is the survey year; *β* the annual time trends in health workers’ compliance at health facility *j* and; *ε_ij_* and μ_*j*_ the residuals at levels 1 and 2, respectively, and capture unobserved variation. A bivariable analysis of each outcome was then performed where the annual trends were fitted as the previous model but were a) adjusted for each covariate at baseline as follows;

$$logit\;(\mu_{ij})=\alpha +\beta 1t_{j}+\beta 2X_{ij}+\varepsilon_{ij}+\mu_{j}$$

where *β1* is the time trends and *β2* is the covariates’ effect at the baseline, and b) each covariate was added as interaction with time (covariate * time) [38] as follows;

$$logit\;(\mu_{ij})=\alpha +\beta 1t_{j}+\beta 2X_{ij}+\beta 3({X*t)}_{ij}+\varepsilon_{ij}+\mu_{j}$$

where *β3* is the interaction between time* covariate effect to evaluate the determinants of time trends in health workers’ compliance. Likelihood ratio tests comparing bivariable with base models at a significance level of a p-value (p) of <0.05 were used to perform the initial selection of covariates and interaction terms for multivariable analysis.

In the multivariable analysis for each outcome, any covariate or interaction term significant at p<0.05 from the bivariable models were included. Covariates and interaction terms that turned non-significant at p<0.05 (based on likelihood ratio test) were dropped from the models. Other potential determinants from the covariates and interaction terms that were non-significant in the bivariable analysis were tested one at a time and added to the model if they satisfied the p<0.05 criterion in the likelihood ratio tests. At the same time, variables that did not lead to a significant change in log-likelihood tests were excluded from the models. The process of adding and dropping covariates and interaction terms was repeated until none of the variables in the model could be omitted without significantly increasing the model log-likelihood, and none of the excluded variables significantly reduced the model log-likelihood. Collinearity between covariates and interactions terms included in the multivariable models was automatically assessed using Stata and collinear variables were omitted when warranted.

The results of the final multivariable models are presented as the adjusted odds ratio (aOR, representing the baseline odds of compliance) and (T-aOR, the adjusted odds ratio of annual trends representing the multiplication factor for odds ratio for the annual change in compliance associated with a unit change in the covariate) with 95% CI. For instance, a T-aOR value of 5.0 for an interaction between age (<5 coded as 0 vs ≥5 years as 1) and time means that the annual increment in the odds of compliance is five times higher for ≥5-year olds compared to under-fives. The adjusted OR from the interaction terms between a covariate and time (T-aOR) were the main results for the determinants of improvement trends adjusting for main effects and covariates at baseline. No multiple comparisons adjustments were conducted [39, 40]. All analyses were conducted using Stata version 15 (StataCorp, College Station, TX, USA).

### Ethics considerations

Ethical approval for the surveys was provided by the Kenyatta National Hospital/University of Nairobi-Ethics and Research Committee (KNH-ERC/R/108). Informed written consent was obtained for all participating health workers, patients, and caretakers of young children.

## Results

### Description of the study population

Frequency distributions of study patients by malaria endemicity zones and ten health facility-, 14 health worker- and six patient-level characteristics are shown for each of the 11 survey rounds in the S1 Table. In summary, a total of 9,173 febrile patients (survey range [SR]: 610–1,241) seen by 1,538 health workers (SR: 116–118) at 1,208 health facilities (SR: 89–143) were analysed. Median patients’ age ranged across surveys between five and eight years. The majority of the patients (>75%) had the main complaint of fever, followed by cough (SR: 42–51%) and headache (SR: 29–45%). Most patients visited government-owned facilities (SR: 75–93%) and dispensaries (SR: 35–63%). At the beginning of the surveys, most patients visited health facilities with only microscopy available, however, over time, this pattern declined (87 to 21%, p<0.001) while facilities with either only RDTs in stock (10 to 51%, p<0.001) or both microscopy and RDTs available increased (4 to 28%, p<0.001). Across all the surveys, drugs were dispensed by nurses or clinical officers ranging between 35 to 55%. A majority of the patients were seen by male health workers (SR: 45–59%) and nurses (SR: 46–70%). The proportion of patients seen by trained health workers increased over time from 0 to 71% (p<0.001) and by supervised health workers from 49 to 70% (p = 0.019). Moreover, health workers’ access to malaria treatment guidelines increased from none to 69% (p<0.001) and Integrated Management of Childhood Illness (IMCI) guidelines from 48 to 74% (p = 0.001). Over three-quarters of the patients were seen by health workers who were knowledgeable about universal testing of all febrile patients across all surveys while those with the correct knowledge of the malaria treatment policy increased from 47 to 95% (p<0.001) (S1 Table).

### Determinants of improvement trends in compliance with overall “test and treat” performance

Overall, the odds of health workers’ compliance with testing and treating patients according to the malaria case-management guidelines increased between 2010 and 2016 by 26% annually (Fig 2, left-most set of bars; OR = 1.26 annually; 95% CI: 1.19–1.33). The results of the unadjusted bivariable analysis of determinants of the annual trends are shown in S2 Table. From the final multivariable model (Table 1), the baseline odds of composite “test and treat” compliance was lower for febrile patients from the lake endemic (aOR = 0.47) compared to low risk zone, those from busy facilities with a caseload of more than 25 febrile patients (aOR = 0.40) compared to less busy facilities, those from facilities with RDTs (aOR = 0.09) compared to microscopy services available and for children aged less than five years (aOR = 0.66) compared to older patients. The annual improvement trends were 67% and 35% higher in lake endemic (p<0.001) and highland epidemic (p<0.001) zones compared to low risk zone. Similarly, the improvements were 15% higher in patients who visited FBO/NGO compared to government-owned facilities (p = 0.036), 46% higher in busy facilities with a caseload of more than 25 febrile patients compared to less busy facilities (p = 0.003), and 49% higher in facilities with RDTs compared to microscopy available on the survey day (p<0.001). Lastly, the improvement trends were 7% higher in under-fives compared to older patients (p = 0.013) (Table 1). None of the health worker level factors, including training, supervision, and access to guidelines, were associated with the improvement trends in overall malaria “test and treat” performance (S2 Table).

**Table 1:** Determinants of improvement trends in compliance with composite malaria “test and treat” performance, 2010–2016—results from the final multivariable model.

Factor	Baseline (2010), % (n/N)	Last survey (2016), % (n/N)	aOR*(95% CI; p-value)	T-aOR** (95% CI; p-value)	P-value for interaction*** (factor*time)
**Year**			0.90 (0.79–1.03; p = 0.117)^a^		
**Epidemiological zone**					
Low risk	28.6 (52/182)	24.5 (24/98)	Ref	Ref	
Lake endemic	27.5 (112/407)	89.6 (206/230)	0.47 (0.25–0.87; p = 0.016)	1.67 (1.43–1.95; p<0.001)	
Coast endemic	25.0 (50/200)	75.0 (60/80)	1.16 (0.54–2.49; p = 0.714)	1.18 (0.97–1.43; p = 0.098)	
Highland epidemic	18.3 (38/208)	62.0 (75/121)	0.55 (0.29–1.03; p = 0.064)	1.35 (1.16–1.58; p<0.001)	
Semi-arid seasonal	40.2 (98/244)	49.0 (71/145)	1.87 (1.02–3.44; p = 0.044)	0.97 (0.84–1.13; p<0.738)	<0.001
** Facility ownership**					
Government	23.7 (219/926)	63.1 (361/572)	Ref	Ref	
FBO/NGO	41.6 (131/315)	73.5 (75/102)	1.24 (0.77–2.00; p = 0.371)	1.15 (1.01–1.30; p = 0.036)	<0.001
**Febrile patients’ caseload**					
≤25 patients	31.4 (277/881)	63.5 (408/643)	Ref	Ref	
>25 patients	20.3 (73/360)	90.3 (28/31)	0.40 (0.20–0.82; p = 0.012)	1.46 (1.14–1.87; p = 0.003)	0.010
**Type of malaria diagnostic tests at the facility**					
Microscopy	30.2 (326/1078)	61.5 (88/143)	Ref	Ref	
RDTs	7.5 (9/120)	64.1 (218/143)	0.09 (0.05–0.18; p<0.001)	1.49 (1.28–1.73; p<0.001)	
Both available	34.9 (15/43)	68.1 (130/191)	1.64 (0.85–3.19; p = 0.143)	0.94 (0.81–1.10; p = 0.467)	<0.001
**Patient age**					
≥5 years	36.3 (235/648)	67.7 (298/440)	Ref	Ref	
<5 years	19.4 (115/593)	59.0 (138/234)	0.66 (0.53–0.82; p<0.001)	1.07 (1.02–1.14; p = 0.013)	0.001

*aOR=fixed effect representing the baseline odds ratio

**T-aOR = Ratio of annual change in the odds between the covariate levels and reference category adjusted for covariates at baseline

^a^ fixed effects of time representing the annual change in the odds of compliance among older patients from the low malaria risk area seen at government-owned, less busy and microscopy only available facilities; The covariates adjusted for at baseline included health worker gender, HW perception of endemicity, supervision with feedback, correct knowledge about “test and treat” policy, temperature, patients’ main complaint of diarrhoea, headache, vomiting, running nose, cough and rash

***the overall p-value for the different time trends across factor categories from likelihood ratio tests

### Determinants of improvement trends in compliance with individual “test and treat” outcomes

Between 2010 and 2016, the overall odds of health workers’ compliance with malaria testing of febrile patients increased by 14% annually (Fig 2, the second-left set of bars; OR = 1.14; 95% CI: 1.07–1.22) while compliance with malaria test results increased annually by 26% (Fig 2, middle set of bars; OR = 1.26; 95% CI: 1.10–1.45) and 81% (Fig 2, second-right set of bars; OR = 1.81; 95% CI: 1.60–2.05) for positive and negative cases, respectively. For the respective outcomes, the results from the unadjusted bivariable analysis of the determinants are presented in S3–S5 Tables.

From the final multivariable model (Table 2), the baseline odds of compliance with malaria testing were lower for febrile patients from facilities with RDTs (aOR = 0.11) compared to microscopy services available and for children aged less than five years (aOR = 0.56) compared to older patients. The annual improvement trends in testing recommendations were twice higher for patients from the lake endemic (p<0.001) and 49% higher in the highland epidemic (p<0.001) zones compared to those from the low risk zone. The annual increase in the odds of testing was also 35% higher in patients visiting facilities stocking only RDTs compared to those providing only malaria microscopy services (p = 0.001). Finally, patient age was also associated with improvements in malaria testing where under-fives compared to older patients had 9% higher annual odds of being tested (p = 0.022) (Table 2).

**Table 2:** Determinants of improvement trends in compliance with malaria testing of febrile patients, 2010–2016—results from the final multivariable model.

Factor	Baseline (2010), % (n/N)	Last survey (2016), % (n/N)	aOR* (95% CI; p-value)	T-aOR** (95% CI; p-value)	P-value for interaction*** (factor*time)
**Year**			0.82 (0.69–0.97; p = 0.019)^a^		
**Epidemiological zone**					
Low risk	39.6 (72/182)	24.5 (24/98)	Ref	Ref	
Lake endemic	43.5 (177/407)	93.9 (216/230)	0.44 (0.18–1.06; p = 0.068)	2.02 (1.63–2.49; p<0.001)	
Coast endemic	40.0 (80/200)	76.3 (61/80)	0.82 (0.28–2.45; p = 0.727)	1.26 (0.97–1.62; p = 0.085)	
Highland epidemic	41.4 (86/208)	70.3 (85/121)	0.50 (0.21–1.17; p = 0.112)	1.49 (1.22–1.83; p<0.001)	
Semi-arid seasonal	45.9 (112/244)	53.1 (77/145)	1.38 (0.60–3.17; p = 0.454)	1.08 (0.89–1.32; p<0.436)	<0.001
**Type of malaria diagnostic tests at the facility**					
Microscopy	43.7 (471/1078)	67.8 (97/143)	Ref	Ref	
RDTs	28.3 (34/120)	67.4 (229/340)	0.11 (0.05–0.25; p<0.001)	1.35 (1.13–1.62; p = 0.001)	
Both available	51.2 (22/43)	71.7 (137/191)	1.51 (0.66–3.50; p = 0.333)	0.93 (0.77–1.13; p = 0.494)	<0.001
** Patient age**					
≥5 years	50.8 (329/648)	71.8 (316/440)	Ref	Ref	
<5 years	33.4 (198/593)	62.8 (147/234)	0.56 (0.41–0.76; p<0.001)	1.09 (1.01–1.18; p = 0.022)	0.022

*aOR = fixed effect representing the baseline odds ratio

**T-aOR = Ratio of annual change in the odds between the covariate levels and reference category adjusted for covariates at baseline

^a^ fixed effect of time representing the annual change in the odds of testing among older patients from the low malaria risk area seen at facilities with microscopy only available; The covariates adjusted for at baseline included facility ownership, facility level, RDT stock-outs, health worker gender, HW perception of endemicity, knowledge about universal testing, temperature, patients’ main complaint of fever, diarrhoea, headache, vomiting, chills, running nose and rash

***the overall p-value for the different time trends across factor categories from likelihood ratio tests.

Only one factor was associated with improvement trends in AL treatment for test positive patients (Table 3). Malaria test positive patients seen by health workers who had access to the new malaria case-management guidelines had lower baseline odds of compliance (aOR = 0.16) and a 50% higher annual increase in the odds of AL treatment compared to those seen by health workers who did not have access to the guidelines (p = 0.027).

**Table 3:** Determinants of improvement trends in compliance with malaria test results, 2010–2016—results from the final multivariable models.

Factor	Baseline (2010), % (n/N)	Last survey (2016), % (n/N)	aOR* (95% CI; p-value)	T-aOR** (95% CI; p-value)	P-value for interaction*** (factor*time)
***1*. *AL treatment for malaria test positive patients***					
**Year**			1.11 (0.88–1.40; p = 0.395)		
**Access to MCM guidelines**					
No	91.3 (178/195)	98.5 (67/68)	Ref	Ref	
Yes	83.3 (5/6)	98.7 (150/152)	0.16 (0.04–0.66; p = 0.011)^a^	1.50 (1.04–2.15; p = 0.027)	0.002
***2*. *No antimalarial treatment for test negative patients***					
**Year**			0.50 (0.34–0.72; p<0.001)^b^		
**Epidemiological zone**					
Semi-arid seasonal	81.0 (34/42)	90.8 (59/65)	Ref	Ref	
Lake endemic	35.6 (26/73)	89.3 (67/75)	0.16 (0.05–0.55; p = 0.004)	1.56 (1.14–2.13 p = 0.006)	
Coast endemic	48.4 (15/31)	96.6 (28/29)	0.46 (0.08–2.58; p = 0.376)	2.50 (1.32–4.72; p = 0.005)	
Highland epidemic	33.3 (21/63)	83.0 (44/53)	0.20 (0.06–0.70; p = 0.012)	1.36 (1.00–1.86; p = 0.053)	
Low risk	56.1 (23/41)	100 (21/21)	0.31 (0.08–1.23; p = 0.096)	2.04 (1.34–3.11; p = 0.001)	<0.001
**Caseload on the survey day**					
≤25 patients	62.3 (104/167)	89.7 (210/234)	Ref	Ref	
>25 patients	40.4 (23/57)	100 (9/9)	0.22 (0.05–0.88; p = 0.032)	2.09 (1.08–4.08; p = 0.030)	0.048
**Retrospective RDT stock-outs**					
Yes	58.5 (120/205)	83.8 (62/74)	Ref	Ref	
No	36.8 (7/19)	92.6 (149/161)	0.23 (0.07–0.83; p = 0.025)	1.51 (1.12–2.02; p = 0.006)	0.022
**Health worker gender**					
Male	61.5 (80/130)	87.4 (111/127)	Ref	Ref	
Female	50.0 (47/94)	93.1 (108/116)	0.55 (0.27–1.13; p = 104)	1.26 (1.03–1.55; p = 0.028)	0.079
**Correct knowledge on targeted treatment policy**					
No	46.8 (51/109)	52.9 (9/17)	Ref	Ref	
Yes	66.1 (76/115)	92.9 (210/226)	0.96 (0.44–2.07; p = 0.910)	1.53 (1.21–1.92; p<0.001)	<0.001
**Fever complaint**					
No	83.2 (108/131)	93.1 (27/29)	Ref	Ref	
Yes	79.0 (94/119)	89.7 (192/214)	0.26 (0.13–0.53; p<0.001)	1.34 (1.09–1.66; p = 0.006)	0.001

*aOR = fixed effect representing the baseline odds ratio

**T-aOR = Ratio of annual change in the odds between the covariate levels and reference category adjusted for covariates at baseline; MCM-malaria case-,management

^a^ fixed effect of time representing the annual change in the odds of AL treatment among test positive patients seen by health workers without access to the guidelines; The covariates adjusted for at baseline in the final model for trends in AL treatment for test positives included in-service training and temperature

^b^ fixed effect for time representing the annual change in the odds of no antimalarial treatment for test negative patients without a main complaint of fever, from semi-arid seasonal transmission areas, seen at less busy facilities, those with retrospective RDTs stock-outs, and seen by male health workers and those not knowledgeable about the targeted treatment policy; The covariates adjusted for at baseline in the final model for trends in no antimalarial treatment for test negatives included the type of diagnostic test, AL stock-outs, facility level, RDT stock-outs, HW perception of endemicity, temperature, patients’ main complaint of headache, vomiting and cough

***the overall p-value for the different time trends across factor categories from likelihood ratio tests.

On the other hand, the improvement trend in health workers’ compliance with no antimalarial treatment for test negative patients was independently associated with six determinants from the final multivariable model (Table 3). The baseline odds of compliance with no antimalarial treatment for test negative patients were lower in lake endemic (aOR = 0.16) and highland epidemic (aOR = 0.20) zones compared to semi-arid seasonal transmission zone, at facilities with a caseload of >25 patients (aOR = 0.22), at facilities with RDT stock-outs (aOR = 0.23) and patients with the main complaint of fever (aOR = 0.26). Compared to patients from semi-arid seasonal transmission areas, patients from the lake endemic (p = 0.006), coast endemic (p = 0.005), and low risk (p = 0.001) zones had higher improvement trends in compliance. At the health facility level, busy facilities with a caseload of >25 febrile patients had a twice higher annual increase in the odds of compliance compared to less busy facilities (p = 0.030). Similarly, patients seen at facilities without historical RDTs stock-outs compared to those with stock-outs had a 51% higher annual increase in the odds of compliance (p = 0.006). At the health worker level, patients seen by females compared to male health workers had a 26% higher annual increase in compliance (p = 0.028). Whereas, when the health workers were knowledgeable about the targeted treatment policy, the annual increase in the odds of compliance was 53% higher (p<0.001). At the patient level, patients with a main complaint of fever had a 34% higher annual increase in the odds of not being treated for malaria when they tested negative (p = 0.006) (Table 3).

### Determinants of improvement trends in compliance with prompt AL administration at the health facility

The overall odds of administration of the first AL dose at the facility increased twice annually between 2010 and 2016 (Fig 2, right-most set of bars; OR = 2.00, 95% CI: 1.66–2.42). S6 Table presents the results of the unadjusted bivariable analysis of determinants of the improvement trend. From the final multivariable model (Table 4), three factors were significantly associated with the improvement trends without significant differences in the baseline odds of compliance. The annual trends in the administration of the first AL dose at the facility were significantly higher in patients from the coast endemic region (p<0.001) and lake endemic (p = 0.020) zones compared to patients from the highland epidemic zone. Moreover, patients who were seen in health facilities where CHWs dispensed medicines had a 90% higher increase in the annual odds of compliance compared to patients having medicines dispensed by higher-level cadres (p = 0.016). Lastly, patients who were seen by health workers who had access to the IMCI guidelines compared to those without access had a 39% higher annual increase in the odds of being given the first AL dose at the health facility (p = 0.026) (Table 4). None of the patient-level factors was significantly associated with the improvement trends in compliance with this task.

**Table 4:** Determinants of improvement trends in compliance with the administration of the first AL dose at the health facility, 2010–2016—results from the final multivariable model.

Factor	Baseline (2010), % (n/N)	Last survey (2016), % (n/N)	aOR* (95% CI; p-value)	T-aOR** (95% CI; p-value)	P-value for interaction*** (factor*time)
**Year**			0.66 (0.41–1.06; p = 0.089)^a^		
**Epidemiological zone**					
Highland epidemic	37.2 (45/121)	27.5 (11/40)	Ref	Ref	
Lake endemic	41.4 (113/273)	69.1 (103/149)	0.47 (0.10–2.26; p = 0.348)	1.67 (1.08–2.59; p = 0.020)	
Coast endemic	14.6 (16/110)	97.1 (34/35)	0.47 (0.05–4.18; p = 0.495)	3.45 (1.75–6.77; p<0.001)	
Semi-arid seasonal	16.2 (24/148)	26.3 (5/19)	0.20 (0.04–1.09; p = 0.062)	1.41 (0.85–2.33; p = 0.180)	
Low risk	20.0 (12/60)	33.3 (1/3)	0.21 (0.03–1.55; p = 0.126)	1.68 (0.86–3.20; p = 0.112)	0.001
**Cadre dispensing drugs**					
Pharmacists	22.4 (32/143)	42.7 (32/75)	Ref	Ref	
Community health workers	21.1 (41/194)	69.8 (30/43)	0.87 (0.12–6.22; p = 0.888)	1.90 (1.13–3.21; p = 0.016)	
Nurse/ Clinician	32.4 (73/225)	73.1 (68/93)	1.95 (0.39–9.76; p = 0.417)	1.33 (0.89–1.98; p = 0.172)	
Others	42.7 (64/150)	68.6 (24/35)	1.26 (0.19–8.26; p = 0.807)	1.52 (0.92–2.53; p = 0.104)	0.149
**Access to IMCI guidelines**					
No	25.9 (94/363)	40.5 (36/89)	Ref	Ref	
Yes	33.2 (116/349)	75.2 (118/157)	0.41 (0.15–1.13; p = 0.085)	1.39 (1.04–1.85; p = 0.026)	0.150

*aOR = fixed effect representing the baseline odds ratio

**T-aOR = Ratio of annual change in the odds between the covariate levels and reference category adjusted for covariate levels at baseline

^a^ fixed effect of time representing the annual change in the odds of first AL dose administration at the facility in highland epidemic areas, when pharmacists dispensed medicines and health workers did not have access to IMCI guidelines; The covariates adjusted for at baseline in the final model included facility ownership, facility level, caseload, HW gender, HW cadre, supervision and temperature

***the overall p-value for the different time trends across factor categories from likelihood ratio tests.

## Discussion

This study is novel in applying regression models and Rowe’s framework [19] to assess the determinants of improvements in health workers’ performance over time using compliance to outpatient malaria diagnosis and treatment guidelines trends. The findings indicate that even when malaria diagnostic tests and recommended antimalarials are available, malaria endemicity and other interventional and non-interventional factors are associated with health workers’ clinical behaviour when the case-management policy is routinely implemented on a large scale over time.

Controlling for other factors, malaria endemicity was independently associated with improvement trends in health workers’ compliance, confirming our previous report on the effect of malaria endemicity on compliance with malaria guidelines [28]. Greater improvements in “test and treat” compliance associated with higher malaria risk zones may be explained by the accumulation of experience with test positive results [41], health workers’ practices considering the pre-test and post-test probability of malaria [42] or the quality improvement activities implemented in these areas [34]. Future qualitative research would help to better understand the effects of endemicity on the compliance patterns observed. The priority for policy implementers should be tailoring of interventions and strategies to improve health worker performance according to the endemicity of the disease and specifically targeting health workers in areas of lower malaria risk.

At the health facility level, the type of diagnostic test, ownership, caseload, and cadre dispensing medicines were determinants of improvement trends in compliance. The type of diagnostic test available at the facility on the survey day, particularly the exclusive availability of RDTs compared to microscopy, was associated with higher improvement trends in testing and subsequently with composite “test and treat” performance. The finding is similar to the reports of interventional studies that indicated that the deployment of RDTs resulted in higher testing rates [43–45]. This is, however, in contrast with our first year (2010) report of higher compliance with testing in facilities with microscopy available compared to RDTs in Kenya [23]. Higher and wider availability of RDTs over time and increased health workers’ trust in the test results due to accumulated field experience [2, 13, 41] may explain the patterns observed. On the contrary, historical RDT stock-outs were associated with lower compliance with negative test results. Both findings support the national policy of deploying RDTs in facilities without diagnostic capabilities (where microscopy is not available) and ensuring the universal and continuous availability of RDTs [46, 47].

Government-owned facilities showed lower improvement trends in “test and treat” compliance compared to FBO/NGO facilities. The higher cost of laboratory services [48], wealthier patients [23], and higher motivation of health workers [49] are possible reasons explaining higher policy adoption in the FBO/NGO sector. Furthermore, at busier facilities, higher improvement trends in compliance with negative test results were found. The results concur with a better quality of care observed for children attending busier facilities in Benin [50] and contrast with a report from Angola [51]. Finally, when CHWs dispensed medicines, they were more likely to provide prompt treatment and administer the first AL dose at the facility over time. This might be because CHWs’ main responsibility is to dispense medicines while higher-level cadres such as pharmacists have broader responsibilities resulting in neglecting the dispensing and counselling tasks. Lay health workers have been shown to improve the quality of care [52–54] and task shifting of the medicine dispensing from often overwhelmed pharmacists to CHWs can be considered by malaria control managers [55].

Several significant associations between health workers’ characteristics and compliance with the guidelines were observed. Interestingly, access to the new malaria case-management guidelines was associated with improvement in AL treatment for test positive patients, a finding in contrast with studies reporting no association between provision of guidelines and recommended ACT treatment [22, 56] or no effect of guideline dissemination on broader health worker performance [57–59]. The finding is, however, in line with reviews suggesting that context-specific dissemination of guidelines can improve care [60, 61]. In this context, health workers’ reference to malaria guidelines may have been of higher interest with respect to treatment than diagnostic changes. Access to IMCI guidelines was associated with improvements in the administration of the first AL dose at the facility. The possible explanation would be that IMCI guidelines put considerable emphasis on dispensing and counselling tasks [62–64].

Additionally, correct health workers’ knowledge about the treatment policy was a predictor of improvement trends in compliance with negative test results. A finding similar to other reports of higher knowledge scores resulting in higher health workers’ compliance [65, 66] and in contrast with other studies indicating that knowledge does not translate to better care [19, 58, 67–70]. The positive association of knowledge with the improvements may indicate that the effect of correct knowledge on compliance is sustained over time, or it is due to other unmeasured factors. Lastly, male health workers showed lower improvements in compliance with negative test results- this factor is rarely examined but contrasts with reports from Uganda reporting no gender association [22, 71, 72].

Conversely, the most widely used interventions to implement case-management policies, (i.e. training and supervision) were not associated with the improvement trends in health workers’ compliance. This contrasted reports from interventional studies that suggested an effect of training and supervision on improving health worker performance [44, 45, 57, 73–77]. The potential explanation for the lack of significant association with improvements over time may be the suboptimal quality of training and supervision implementation that affected the effectiveness of these strategies [78, 79] or possible contamination of patient observations as frontline health workers who attend the training are meant to mentor on-job other health workers at their facilities passing the correct information to untrained health workers [23]. Another reason could relate to the intention of in-service training to expand knowledge, while supervision may increase health worker motivation and translation of the knowledge into practice. However, this might be inadequate for a long-term effect on health worker performance as their influence decrease [80–82] or do not change over time [83]. This calls for further qualitative research to understand the details of the quality and content of training and supervision routinely delivered to health workers and interventional studies of the most cost-effective set of strategies to improve health worker performance further.

At the patient level, only two factors were associated with improvements, patient age and the main complaint of fever. Patient age was key in improvement in compliance with testing, which was higher in under-five children compared to older patients. This is likely to reflect the adoption of malaria testing after 2010 among under-fives, the patient population that was presumptively treated before 2010 [3]. Finally, patients’ main complaint of fever was associated with improvement in compliance with the negative test results. Over time, health workers might have acknowledged that fevers cannot be equated with malaria due to the decline in transmission in most areas hence better compliance when patients reported fever as the main complaint [84].

The study has some limitations. The sample sizes might have been small to allow for the detection of the effect of some factors. Moreover, health workers’ behaviour might be affected by a variety of factors, including contextual and latent ones like motivation, attitude, experience, and confidence, that could not be examined from the data and require further qualitative research. Also, we performed multiple comparisons of factors, and some of the results may have been significant by chance. Finally, the performance of some of the outcomes plateaued from the eighth round, suggesting a need for separately exploring the specific determinants of compliance during this recent period.

## Conclusion

This study revealed a series of factors associated with improvement trends in health workers’ compliance with outpatient malaria case-management guidelines over time at health facilities with malaria “test and treat” commodities in Kenya. The improvements in clinical practices examined over six years were associated with high malaria risk areas, RDT availability, high patient caseloads, female and non-governmental health workers, dissemination of malaria and IMCI guidelines, under-five patients’ age, the main complaint of fever and health workers knowledge about the targeted treatment policy. Therefore, targeting of low malaria risk areas, facilities with low caseloads, male and government health workers, continuous availability of RDTs, improved health workers’ knowledge about the policy considering age and fever, and dissemination of malaria and IMCI guidelines might improve health workers’ compliance with malaria guidelines. For prompt treatment and administration of the first AL dose at the facility, task-shifting duties to CHWs can be considered. Further qualitative research to understand the details of the quality, content and delivery of training and supervision routinely delivered to health workers and interventional studies of the most cost-effective set of strategies to further improve outpatient malaria case-management performance are needed.

## Supporting information

S1 Appendix Exit interview form. (PDF)

S2 Appendix Health facility assessment form. (PDF)

S3 Appendix Health worker interview form. (PDF)

S1 Table Description of patients by malaria endemicity and health facility-, health worker- and patient-level characteristics by survey round. (DOCX)

S2 Table Bivariable analysis of determinants of improvement trends in compliance with overall “test and treat” performance, 2010–2016. *1-main effects estimate adjusting for time; 2- T-OR = unadjusted odds ratio from the covariate and time interaction; FBO/NGO- Faith-based organisation/Non-Governmental organisation; RDT-rapid diagnostics tests; AL-artemether-lumefantrine; IQR-interquartile range; HW-health worker; MCM-malaria case-management.
(DOCX)

S3 Table Bivariable analysis of determinants of improvement trends in compliance with malaria testing of febrile patients, 2010–2016. *1-main effects estimate adjusting for time; 2- T-OR = unadjusted odds ratio from the covariate and time interaction; FBO/NGO- Faith-based organisation/Non-Governmental organisation; RDT-rapid diagnostics tests; AL-artemether-lumefantrine; IQR-interquartile range; HW-health worker; MCM-malaria case-management.
(DOCX)

S4 Table Bivariable analysis of determinants of improvement trends in compliance with AL treatment for malaria test positive patients, 2010–2016. *1-main effects estimate adjusting for time; 2- T-OR = unadjusted odds ratio from the covariate and time interaction; FBO/NGO- Faith-based organisation/Non-Governmental organisation; RDT-rapid diagnostics tests; AL-artemether-lumefantrine; IQR-interquartile range; HW-health worker; MCM-malaria case-management.
(DOCX)

S5 Table Bivariable analysis of determinants of improvement trends in compliance with no antimalarial treatment for test negative patients, 2010–2016. *1-main effects estimate adjusting for time; 2- T-OR = unadjusted odds ratio from the covariate and time interaction; FBO/NGO- Faith-based organisation/Non-Governmental organisation; RDT-rapid diagnostics tests; AL-artemether-lumefantrine; IQR-interquartile range; HW-health worker; MCM-malaria case-management.
(DOCX)

S6 Table Bivariable analysis of determinants of improvement trends in compliance with the administration of first AL dose at the facility, 2010–2016. *1-main effects estimate adjusting for time; 2- T-OR = unadjusted odds ratio from the covariate and time interaction; FBO/NGO- Faith-based organisation/Non-Governmental organisation; RDT-rapid diagnostics tests; AL-artemether-lumefantrine; IQR-interquartile range; HW-health worker; MCM-malaria case-management.
(DOCX)
