# Supplementary material for: Determinants of improvement trends in health workers’ compliance with outpatient malaria case-management guidelines at health facilities with available “test and treat” commodities in Kenya
Source: PLoS One. 2021 Nov 5;16(11):e0259020. doi: 10.1371/journal.pone.0259020 (PMC8570506; doi:10.1371/journal.pone.0259020)
Supplement: S1 Appendix — (PDF) [file pone.0259020.s001.pdf]

National Malaria Control Program, Ministry of Health
Malaria OPD case management survey – Exit interview form

P HF HW PAT

ID Number..[__]-[__|__]-[__]-[__|__]

Date..[__|__] [__|__] [__|__]

Name of county..[_______________________________]

Name of sub-county....................................[_______________________________]

Name of health facility...............................[_______________________________]

Name of health worker...............................[_______________________________]

Name of data collector...............................[_______________________________]

1. Rapid screening

a. Was patient **referred** to another facility for hospitalisation? (Y/N) [Check card, ask].............. [__]

b. Was patient **admitted** to this facility for hospitalisation? (Y/N) [Check card, ask] .... [__]

c. Is this patient's **follow up visit** for the same illness? (Y/N) [Check card, ask].......................... [__]

d. Is patient's **weight less than 5 kg**? (Y/N) [Observe, check card, measure] .... [__]

e. Is patient presenting **without fever** during this illness? (Y/N) [Check card, ask] .... [__]

f. Is patient **likely to be pregnant**? (Y/N) [Observe, check card, ask] .... [__]

If YES to any of the above questions do not proceed with the interview

2. History and measurements

a. Patient's **age**? (years-months) [Check card, ask]..[__|__]-[__|__]

b. Patient's **sex**? (M/F) [Observe, ask] .... [__]

c. Patient's **weight** in kg? (one decimal point) [Check card, measure] .... [_____|__]

d. Patient's **temperature** in °C? (one decimal point) [Check card, measure]....................... [_____|__]

e. For **how many days** patient was sick? (Today = 1) [Ask]...[___]

f. Does the patient's **present illness** involve a **fever**? (Y/N) [Check card, ask]............................ [__]

g. Was fever present **in last 48 hours**? (Y/N) [Check card, ask].. [__]

h. How many **illness episodes with fever** in past 1 month? [Ask]...[___]

[__]-[__|__]-[__]-[__|__]

i. Patient's main **complaints**? [Ask without prompting & enter all complaints reported]

Complaint 1 .... [____]
Complaint 2 .... [____]
Complaint 3 .... [____]
Complaint 4 .... [____]
Complaint 5 .... [____]
Complaint 6 .... [____]
Complaint 7 .... [____]

j. Did patient take any **antimalarial** for this illness **PRIOR** to this visit?(Y/N) [If No go to Q3] [__]
If Yes,

Name & formulation of the **last** antimalarial?.................. [__________________________]

When was the **first dose** taken? (Today = 1)..[_____]

When was the **last dose** taken? (Today = 1)..[_____]

Number of **doses taken** in total? ....[_____]

Number of **tablets/spoons taken** in total?..[_____]

IF more than one antimalarial was taken fill the following section for the preceding one

Name & formulation of the **preceding** antimalarial?........ [__________________________]

When was the **first dose** taken? (Today = 1)..[_____]

When was the **last dose** taken? (Today = 1)..[_____]

Number of **doses taken** in total? ....[_____]

Number of **tablets/spoons taken** in total?..[_____]

Fill the following question ONLY for patients in Nairobi County

k. Has patient travelled **outside of Nairobi in past 30 days**?(Y/N) [If No go to Q3] .... [__]

If Yes, to which district did the patient travel? [ask] ....[______________________________]

3. Routine health workers practices

a. Did any health worker **ask/record patient's age** during this visit? (Y/N) [Check card, ask] .... [__]

b. Did any health worker **measure weight**? (Y/N) [Check card, ask] .... [__]

c. Did any health worker **measure temperature**? (Y/N) [Check card, ask] .... [__]

d. Did any health worker **ask about previous use of antimalarials**? (Y/N) [Check card, ask] .... [__]

[__]- [__|__]-[__]-[__|__]

4. Laboratory

a. Was the patient sent **for malaria blood slide**? (Y/N) [Check card, ask] [If No go to Q4b]............ [__]

If Yes, was **malaria blood slide performed**? (Y/N) [Check card, ask].............................. [__]

b. Did patient have malaria **RDT performed**? (Y/N) [Check card, ask]...................................... [__]

c. **Laboratory report**? [Rewrite full report of all laboratory investigations requested, performed and results reported exactly as it is written in the card; if there is no lab report write NONE in the box]

5. Diagnosis and treatment

a. Patient's **diagnosis**? [Rewrite all diagnoses exactly as it is written in the patient's card; if there is no diagnosis write NONE in the box]

b. **Treatment** prescribed? [Rewrite full prescriptions for all treatments exactly as it is written in the patient's card; if there is no treatment prescribed write NONE in the box]

6. Antimalarial drug dispensing [Complete this section **only if ORAL antimalarial drug** was prescribed]

Identify **ORAL ANTIMALARIAL** drug in the prescription! Ask to **see** drugs!

a. **Name & formulation** of **oral** antimalarial?...............[_______________________________]

b. Was the drug **dispensed** to the patient/caretaker at the facility? (Y/N) [Ask, check drugs] . [__]

c. Was the **first dose administered** at facility? (Y/N) [Ask, check drugs].............................. [__]

d. Was the **first dose swallowed in front** of any health worker? (Y/N) [Ask] .... [__]

e. Did any of HWs **explain you how to give/take** drug at home? (Y/N) [Ask] .... [__]

f. Did any of HWs **tell you to give/take** the second dose **after 8 hours**? (Y/N) [Ask]......... [__]

g. Did any of HWs tell you to give/take drug **after meal or with food**? (Y/N) [Ask].......... [__]

h. Were you told to **complete all doses** even if you/your child feels better? (Y/N) [Ask].... [__]

i. Were you advised what to do in case of **vomiting**? (Y/N) [Ask] .... [__]

If **Yes**, what were you advised?

j. Were you advised what to do in case of **drug reactions**? (Y/N) [Ask]............................. [__]

IF more than one oral antimalarial is prescribed fill the following section for 2nd antimalarial

Identify **second ORAL ANTIMALARIAL** drug in the prescription! Ask to **see** drugs!

a. **Name & formulation** of **oral** antimalarial?...............[______________________________]

b. Was the drug **dispensed** to the patient/caretaker at the facility? (Y/N) [Ask, check drugs] . [___]

c. Was the **first dose administered** at facility? (Y/N) [Ask, check drugs]............................. [___]

d. Was the **first dose swallowed in front** of any health worker? (Y/N) [Ask] .... [___]

e. Did any of HWs **explain you how to give/take** drug at home? (Y/N) [Ask] .... [___]

f. Did any of HWs **tell you to give/take** the second dose **after 8 hours**? (Y/N) [Ask]......... [___]

g. Did any of HWs tell you to give/take drug **after meal or with food**? (Y/N) [Ask].......... [___]

h. Were you told to **complete all doses/finish the course**? (Y/N) [Ask] .... [___]

i. Were you advised what to do in case of **vomiting**? (Y/N) [Ask] .... [___]

If **Yes**, what were you advised?

[ ]

j. Were you advised what to do in case of **drug reactions**? (Y/N) [Ask]............................. [___]

7. Drug dispensing of AL [complete this section **ONLY for patients with dispensed AL**]

a. Was patient given **ORIGINAL, not cut AL** pack(s)?(Y/N) [Check pack] [If No go to Q7b] ...... [___]

If Yes, **which blister pack(s)** was given and **how many of each** was given?

	**Coartem (number)**	**Artefan (number)**	**Coartem-D (number)**	**Co-falcinum (number)**
AL 6 tabs pack				
AL 12 tabs pack				
AL 18 tabs pack				
AL 24 tabs pack				

b. Was patient given any **CUT AL blister** pack(s)? (Y/N) [Check pack] [If No go to Q7c]............ [___]

If Yes, describe **which pack** was cut and how was AL dose dispensed?

[ ]

c. Was patient given any **loose AL tablets**? (Y/N) [Check drugs].. [___]

d. Fill this section only for patients with dispensed **Coartem-D**? (Y/N) [Check drugs]

Was first dose of Coartem D **administered at health facility**? (Y/N) [Ask, check drugs] .... [___]

If **Yes**, was Coartem D administered **dispersed in the water**? (Y/N) [Ask] .... [___]

Was mother **instructed** to give Coartem D at **home dispersed** in the water? (Y/N) [Ask] [___]
