# Supplementary material for: Determinants of improvement trends in health workers’ compliance with outpatient malaria case-management guidelines at health facilities with available “test and treat” commodities in Kenya
Source: PLoS One. 2021 Nov 5;16(11):e0259020. doi: 10.1371/journal.pone.0259020 (PMC8570506; doi:10.1371/journal.pone.0259020)
Supplement: S2 Appendix — (PDF) [file pone.0259020.s002.pdf]

National Malaria Control Program, Ministry of Health
Malaria OPD case management survey – Health facility assessment

P HF

ID Number..[__] - [__|__]

Date..[__|__] [__|__] [__|__]

Name of county..[______________________________]

Name of sub-county...................................[______________________________]

Name of health facility...............................[______________________________]

Name of data collector...............................[______________________________]

1. Basic health facility infrastructure

a. Does the health facility have **electricity** today? (Y/N).. [__]

b. Is any **water** available at health facility today? (Y/N) [If No go to Q1c]................................. [__]

If Yes, **source** of the water? [Check all that apply]

Running water at the facility? (Y/N) .... [__]

Pumped water at the facility (e.g. borehole)? (Y/N)... [__]

Rainfall collection from the water tank? (Y/N) .... [__]

Water brought in from **outside** of the facility? (Y/N) .... [__]

If Yes, the cost of 20 liters? (number in KSh) ....[________]

Other source (specify)? (Y/N) .... [________________________][__]

c. Is there a functioning **weighing scale** at the OPD of health facility? [Check all that apply]

Hanging Salter scale? (Y/N) .... [__]

Infant scale? (Y/N)... [__]

Bathroom scale? (Y/N) .... [__]

Adults scale? (Y/N)... [__]

Others (specify)? (Y/N) .... [________________________][__]

d. Is there at least one functioning **thermometer** at the OPD of health facility? (Y/N) .... [__]

e. Is there a **mobile phone network** at this health facility? (Y/N).. [__]

f. Name of **contact HW** for follow up calls on stocks .... [___________________________]

Mobile **phone number(s)** of the contact HW........... [___________________________]

g. Name of **alternative HW** for follow up calls on stocks...... [___________________________]

Mobile **phone number(s)** of the alternative HW ...... [___________________________]

2. Guidelines and wall charts

a. Is there a facility copy of **2006 or 2008 malaria guideline** for HWs [Show example]? (Y/N). [__]

b. Is there a copy of **2010, 2012 or 2014 malaria guideline** for HWs [Show example]? (Y/N)... [__]

c. Is there a facility copy of **IMCI guideline** for HWs [Show example]? (Y/N) .... [__]

d. Is there a facility copy of **malaria management chart booklet** [Show example]? (Y/N)........ [__]

e. Is there a facility copy of **Coartem-D health workers workbook** [Show example]? (Y/N).... [__]

f. Is there a facility copy of **malaria user's guide for laboratory**? (Y/N).............................. [__]

g. Are the following malaria **wall charts exposed** at the facility [Check examples]?

Algorithm for assessing and treating children <5 yrs with fever? (Y/N) .... [__]

AL dispensing procedure and dosing schedule? (Y/N) .... [__]

Malaria outpatient algorithm for older children and adults? (Y/N)............................... [__]

Malaria outpatient algorithm for children and adults (**new chart**)? (Y/N) .... [__]

3. OPD clinical staffing and relevant case management training

a. **How many** of the following **health workers** perform **outpatient** consultations, and how many **of these** have received **training on malaria case management (CM), use of RDTs and IMCI**?

	All HWs (number)	Malaria CM trained since 2010 (number)	Malaria CM trained 2006-2009 (number)	RDT trained (number)	IMCI trained (number)
**Doctors**					
**Clinical officers**					
**Nurses**					
**CHW**					
**Others (specify):**					
**Others (specify):**					

4. Laboratory staffing and relevant in-service training

a. **How many** of the following **laboratory health workers** perform **malaria testing** and how many **of these** have received **in-service training on malaria microscopy** (since 2006) and **RDT use**?

	All HWs (number)	Malaria microscopy trained (number)	RDT trained (number)
**Lab technologists**			
**Lab technicians**			
**Others (specify):**			

b. What cadre is performing **malaria microscopy today**? .... [______________________]

c. What cadre is performing **malaria RDTs today**? .... [______________________]

5. Drug dispensing staffing and in-service training

a. **How many** of the following **health workers** dispense **antimalarial drugs** and how many **of these** have received **in-service training on antimalarial drug management**?

	All HWs (number)	Trained on management of malaria medicines (number)
**Pharmacy technologists**		
**Pharmacists**		
**Nurses**		
**CHWs**		
**Others (specify):**		

b. What cadre is **dispensing** drugs **today**? .... [______________________]

6. Supervision

a. Has facility had **any supervisory visit in past 3 mths** (Mar-May)? (Y/N) [If No go to Q6b]... [___]

If Yes, was **malaria case management topic of** any visit? (Y/N) [If No go to Q6b] .... [___]

If Yes,

What is **source?** (KEPI book, visitors book, verbal).[______________________]

Was outpatient **malaria case management** observed? (Y/N) .... [___]

If Yes, by who? (title).................................. [______________________]

b. Has facility had any quality control visit of **malaria microscopy**? (Y/N)............................ [___]

If Yes, by who? (title)... [______________________]

c. Has facility had any supervisory visit on **malaria RDT** use? (Y/N)..................................... [___]

If Yes, by who? (title)... [______________________]

d. Has facility had any supervisory visit on **drug management**? (Y/N) .... [___]

If Yes, by who? (title)... [______________________]

[__]-[__|__]

7. Availability of malaria diagnostic services

a. Is malaria **microscopy routinely provided** at health facility? (Y/N) .... [__]

If Yes, is malaria **microscopy** service functional **today**? (Y/N)....................................... [__]

b. Availability of **malaria RDTs today**? [If different RDT tests separate information by product]

	Non-expired quantity? (number of tests)	Expired quantity? (number of tests)
**RDT 1 (write test name):**		
**RDT 2 (write test name):**		

c. **Source of RDTs** for health facility? .... [________________________________]

8. Availability of AL and malaria medicines inventory materials on survey day

a. Availability of **AL** on survey day?

	Non-expired quantity [physical count]			Non-expired quantity [record count]			Expired quantity [physical count]
	Store	Dispensing area	Total	Stock card	AL register	Total	
AL 6 pack [No of blisters]							
AL 12 pack [No of blisters]							
AL 18 pack [No of blisters]							
AL 24 pack [No of blisters]							
Coartem **D** 6 pack [No of blisters]							
Coartem **D** 12 pack[No of blisters]							

b. Is drug **stock/bin card** available at HF? (Y/N) .... [__]

If Yes, is it regularly updated [check for last one month]? (Y/N) .... [__]

c. Is **DAR/AL dispenser's book** available at HF? (Y/N) .... [__]

If Yes, is it regularly updated [check for last one month]? (Y/N) .... [__]

d. Is **monthly summary form for malaria medicines** available at HF? (Y/N)........................ [__]

If Yes, is it regularly completed [check for last 3 months]? (Y/N) .... [__]

e. Is **ADR form (yellow form)** available at HF? (Y/N) .... [__]

f. Is **poor quality medicinal product reporting form (pink form)** available at HF? (Y/N)... [__]

9. Availability of other antimalarials on survey day [do physical count]

	Non-expired quantity?	Expired quantity?
Chloroquine tablets [No of tablets]		
Chloroquine syrup [No of liters]		
Chloroquine injections [No of vials]		
SP tablets [No of tablets]		
SP syrup or drops [No of bottles]		
Amodiaquine tablets [No of tablets]		
Amodiaquine syrup [No of liters]		
Quinine tablets [No of tablets]		
Quinine injections [No of vials]		
Artesunate injection [No of vials]:		
Other AM (write name):		

[__]-[__|__]

10. Availability of NON-EXPIRED medicines in STORE on survey day

Name of antibiotic	Available (Y/N)	Name of antibiotic	Available (Y/N)
Cotrimoxazol tab		Tetracycline tablets	
Cotrimoxazol syrup		Doxycycline capsules	
Amoxycillin tabs/capsules		Metronidazol tab	
Amoxycillin syrup		Metronidazol syrup	
Ceftriaxone injection		Albendazol tab	
Ciprofloxacin tablets		Chloramphenicol capsules	
Erythromycin tablets		Chloramphenicol syrup	
Kanamycin injection		Chloramphenicol injection	
Procaine penicillin injection		Benzylpenicillin injection	
Tetracycline Eye Ointment		Gentamycin injection	
Paracetamol tab		Chlorpheniramine tab	
Adrenaline inj		Hydrocortisone inj	
ORS Sachets		Clotrimazole cream	
Loperamide tabs		Zinc Sulphate	
Nystatine susp bottle		Magnesium sulphate	
Other AB (write name):		Other AB (write name):	

11. Quantities of AL ordered and received

a. Name of regular **AL supplier**?

KEMSA? (Y/N) .... [__]

MEDS? (Y/N) .... [__]

Others (specify)? (Y/N) .... [______________________________][__]

b. **Date of last AL delivery** to the health facility? [check delivery note]......[__|__] [__|__] [__|__]

c. Does the facility function on **pull** system - **ordering AL**? (Y/N) [If No go to Q11d]................ [__]

If Yes, enter the quantities of AL ordered and quantities of AL received **for the last order**:

	Quantity ordered? (number)	Quantity received? (number)
AL 6 tabs pack		
AL 12 tabs pack		
AL 18 tabs pack		
AL 24 tabs pack		

Date of AL order preceding last AL delivery? [check SOF] ....[__|__] [__|__] [__|__]

d. Does the facility function on **push** system – **not ordering AL**? (Y/N) [If No go to Q12]......... [__]

If Yes, enter the most recent quantities of **AL received**:

	Quantity received? (number)
AL 6 tabs pack	
AL 12 tabs pack	
AL 18 tabs pack	
AL 24 tabs pack	

Date of AL delivery preceding last AL delivery? [check delivery note][__|__] [__|__] [__|__]

[__]-[__|__]

12. STOCK-OUTs in past 3 months [for each non-expired AL preparation, tick the **day out of stock** in each evaluation month and calculate **Total number of stock-out days** over 3 months period; if stock-out day information is not available from AL record books enter NA in the box and **do not count** total number of stock-out days]**.**

AL 6 tablet pack	1	2	3	4	5	6	7	8	9	10	11	12	13	14	15	16	17	18	19	20	21	22	23	24	25	26	27	28	29	30	31	Total
March																																
April																																
May																																
**AL 12 tablet pack**	1	2	3	4	5	6	7	8	9	10	11	12	13	14	15	16	17	18	19	20	21	22	23	24	25	26	27	28	29	30	31	Total
March																																
April																																
May																																
**AL 18 tablet pack**	1	2	3	4	5	6	7	8	9	10	11	12	13	14	15	16	17	18	19	20	21	22	23	24	25	26	27	28	29	30	31	Total
March																																
April																																
May																																
**AL 24 tablet pack**	1	2	3	4	5	6	7	8	9	10	11	12	13	14	15	16	17	18	19	20	21	22	23	24	25	26	27	28	29	30	31	Total
March																																
April																																
May																																
**All four AL packs**	1	2	3	4	5	6	7	8	9	10	11	12	13	14	15	16	17	18	19	20	21	22	23	24	25	26	27	28	29	30	31	Total
March																																
April																																
May																																
**SP tablets**	1	2	3	4	5	6	7	8	9	10	11	12	13	14	15	16	17	18	19	20	21	22	23	24	25	26	27	28	29	30	31	Total
March																																
April																																
May																																
**QN tablets**	1	2	3	4	5	6	7	8	9	10	11	12	13	14	15	16	17	18	19	20	21	22	23	24	25	26	27	28	29	30	31	Total
March																																
April																																
May																																
**QN injection**	1	2	3	4	5	6	7	8	9	10	11	12	13	14	15	16	17	18	19	20	21	22	23	24	25	26	27	28	29	30	31	Total
March																																
April																																
May																																
**Artesunate inj**	1	2	3	4	5	6	7	8	9	10	11	12	13	14	15	16	17	18	19	20	21	22	23	24	25	26	27	28	29	30	31	Total
March																																
April																																
May																																

[__]-[__|__]

13. ABSENCE of malaria microscopy service and RDT STOCK-OUT in past 3 months [for each evaluation day, tick the **box if malaria microscopy service was not provided or RDTs were out of stock** and calculate **Total number of absence or stock-out days** over the whole 3 months period; if stock-out day information is not available from laboratory books enter NA in the box and **do not** count total number of stock-out days]

Malaria microscopy absent	1	2	3	4	5	6	7	8	9	10	11	12	13	14	15	16	17	18	19	20	21	22	23	24	25	26	27	28	29	30	31	Total
March																																
April																																
May																																

Malaria RDT stock-out	1	2	3	4	5	6	7	8	9	10	11	12	13	14	15	16	17	18	19	20	21	22	23	24	25	26	27	28	29	30	31	Total
March																																
April																																
May																																

Both diagnostics absent	1	2	3	4	5	6	7	8	9	10	11	12	13	14	15	16	17	18	19	20	21	22	23	24	25	26	27	28	29	30	31	Total
March																																
April																																
May																																

14. Stock out of antimalarial drugs and absence of diagnostics for 7 consecutive days in past 3 months

a. Were the following drugs, diagnostic tests or services out of stock or absent for **at least 7 consecutive days in past 3 months** (March-May)?

	**According to record review? (Y/N/NA)**	**If Not available (NA) in records, according to health worker's report? (Y/N/DK)**
Artemether-lumefantrine 6 tablets pack		
Artemether-lumefantrine 12 tablets pack		
Artemether-lumefantrine 18 tablets pack		
Artemether-lumefantrine 24 tablets pack		
All four Artemether-lumefantrine packs		
SP tablets		
Quinine tablets		
Quinine injections		
Artesunate injection		
Malaria RDT		
Malaria microscopy		
Both malaria diagnostics (RDT and microscopy)		
