# Supplementary material for: Determinants of improvement trends in health workers’ compliance with outpatient malaria case-management guidelines at health facilities with available “test and treat” commodities in Kenya
Source: PLoS One. 2021 Nov 5;16(11):e0259020. doi: 10.1371/journal.pone.0259020 (PMC8570506; doi:10.1371/journal.pone.0259020)
Supplement: S3 Appendix — (PDF) [file pone.0259020.s003.pdf]

National Malaria Control Program, Ministry of Health
Malaria OPD case management survey – Health worker interview

P HF HW

ID Number..[__]-[__|__]-[__]

Date.. [__|__] [__|__] [__|__]
Name of county.. [_______________________________________]
Name of sub-county.. [_______________________________________]
Name of health facility.. [_______________________________________]
Name of health worker.. [_______________________________________]
Name of interviewer.. [_______________________________________]

1. Background characteristics

a. Health worker's **age**? (years) .... [__|__]
b. Health worker's **sex**? (M/F) ....[__]
c. What is the health worker's **cadre**?
Clinical officer? (Y/N)..[__]
Nurse? (Y/N)...[__]
Community Health Worker? (Y/N) ....[__]
Others (specify)? (Y/N) .... [_______________________________][__]
d. Are you the facility **in-charge**? (Y/N)..[__]

2. In-service training related to malaria [If HW trained more than once, enter details for the most recent training]

a. Have you ever attended **IMCI** training? (Y/N) [If No go to Q2b]...[__]
If Yes, **date** of training? (month-year).. [__|__]-[__|__]
Was use of **AL** part of the IMCI course? (Y/N)...[__]
Was use of **RDTs** part of the IMCI course? (Y/N) ....[__]
b. Have you attended **malaria** case management training that **included AL use**? (Y/N) [If No go to Q2c] ..[__]
If Yes, **date** of training? (month-year).. [__|__]-[__|__]
Organization giving the course? (name)........ [_______________________________]
Course venue? (town and setting)................. [_______________________________]
Duration of training? (number of days) .... [___]
Participants? (number) .... [___]
Clinical practice included? (Y/N)...[__]
Was **use of RDTs** part of the course? (Y/N)...[__]
c. Have you ever attended **RDT specific malaria** training? (Y/N) [If No go to Q3a]..................................[__]
If Yes, **date** of training? (month-year).. [__|__]-[__|__]
d. Have you ever been trained/oriented how to use **injectable Artesunate**? (Y/N) [If No go to Q3] ....[__]
If Yes, was this done during any of these trainings/orientations?[prompt all responses]
3 days malaria case management training? (Y/N)...[__]
1 day training on severe malaria management? (Y/N)..[__]
ETAT training? (Y/N)..[__]
IMCI training? (Y/N) ....[__]
On-job training/orientation? (Y/N)...[__]
Others (specify)? (Y/N)............................ [_______________________________][__]

3. Guidelines

a. Do you have access to **2006 or 2008 malaria guideline** for HWs [Show example]? (Y/N) .... [__]

b. Do you have access to **2010, 2012 or 2014 malaria guideline** for HWs [Show example]? (Y/N) .... [__]

c. Do you have access to **malaria management chart booklet** [Show example]? (Y/N)............................. [__]

d. Do you have access to **IMCI guideline** booklet [Show example]? (Y/N) .... [__]

4. Supervision

a. Did you have **any supervisory visit** in the **last 3 months** (March-May)? (Y/N) [If No go to Q5a] .... [__]

If Yes,

Was **malaria case management topic of** any of these visits? (Y/N) [If No go to Q5a] .... [__]

If Yes, **how many** such visits you had in last 3 months (March-May)? (number)... [___]

What did these visits **include related to malaria case management**? [Prompt all responses]

Review of **malaria records** and registers? (Y/N)... [__]
Discussion with supervisor on malaria case management? (Y/N)..................... [__]
Observation of outpatient consultations? (Y/N) .... [__]
Provision of **feedback**? (Y/N)... [__]
Other component (specify)? (Y/N)................ [________________________][__]
Other component (specify)? (Y/N)................ [________________________][__]

5. Knowledge about malaria case management policies

a. Classify following statements according to national recommendations for **use and interpretation of malaria test** in febrile, non-severe patients presenting for an initial outpatient visit at facilities where microscopy or RDTs are available? [Allow health worker to see statements and ask him to classify each statement as true, false or "don't know"]

All patients with fever or history of fever should be tested for malaria? (T/F/DK) .... [___]
Only patients who test positive should be treated for malaria? (T/F/DK) .... [___]

b. Would you classify this area as **high or low** malaria risk area? (H for high / L for low)......................... [___]

c. What is the **name of the first line** drug recommended for treatment of **uncomplicated malaria**?
[Write health workers' **responses for each category;** only **one response** allowed per category]

Children **above 5 kg and adults**?... [__________________________]
Children **below 5 kg**? .... [__________________________]
Pregnant women in **first** trimester?... [__________________________]
Pregnant women in **second & third** trimester?... [__________________________]

d. What is the **second line** drug recommended for treatment of **uncomplicated malaria**? [only **one response**]
.... [__________________________]

e. What is the **first line** drug recommended for treatment of **severe malaria**? [only **one response**]
.... [__________________________]

6. Pharmacovigilance

a. Have you ever **reported adverse drug reaction** on antimalarial drugs [yellow form]? (Y/N) .... [__]

If No, why not?... [_______________________________________]

b. Have you ever **reported poor quality antimalarial** product [pink form]? (Y/N) .... [__]

If No, why not?... [_______________________________________]
