# Supplementary material for: Determinants of improvement trends in health workers’ compliance with outpatient malaria case-management guidelines at health facilities with available “test and treat” commodities in Kenya
Source: PLoS One. 2021 Nov 5;16(11):e0259020. doi: 10.1371/journal.pone.0259020 (PMC8570506; doi:10.1371/journal.pone.0259020)
Supplement: S1 Table — (DOCX) [file pone.0259020.s004.docx]

| **Survey round** | **1**  **N=1241**  **n (%)** | **2**  **N=862**  **n (%)** | **3**  **N=634**  **n (%)** | **4**  **N=738**  **n (%)** | **5**  **N=905**  **n (%)** | **6**  **N=1270**  **n (%)** | **7**  **N=771**  **n (%)** | **8**  **N=741**  **n (%)** | **9**  **N=727**  **n (%)** | **10**  **N=610**  **n (%)** | **11**  **N=674**  **n (%)** |
| --- | --- | --- | --- | --- | --- | --- | --- | --- | --- | --- | --- |
| **Epidemiological zone**  Lake endemic  Coast endemic  Highland epidemic  Semi-arid seasonal  Low risk | 407 (32.8)  200 (16.1)  208 (16.8)  244 (19.7)  182 (14.7) | 275 (31.9)  95 (11.0)  213 (24.7)  195 (22.6)  84 (9.7) | 161 (25.4)  76 (12.0)  185 (29.2)  133 (21.0)  79 (12.5) | 193 (26.2)  62 (8.4)  127 (17.2)  218 (29.5)  138 (18.7) | 325 (35.9)  41 (4.5)  175 (19.3)  183 (20.2)  181 (20.0) | 355 (28.0)  110 (8.7)  262 (20.6)  272 (21.4)  271 (21.3) | 162 (21.0)  78 (10.1)  152 (19.7)  207 (26.7)  172 (22.3) | 270 (36.4)  72 (9.7)  150 (20.2)  189 (25.5)  60 (8.1) | 185 (25.5)  33 (4.5)  221 (30.4)  159 (21.9)  129 (17.7) | 203 (33.3)  85 (13.9)  84 (13.8)  126 (20.7)  112 (18.4) | 230 (34.1)  80 (11.9)  121 (18.0)  145 (21.5)  98 (14.5) |
| **Health Facility level** | | | | | | | | | | | |
| **Facility ownership**  FBO/NGO  Government | 315 (25.4)  926 (74.6) | 99 (11.5)  763 (88.5) | 113 (17.8)  521 (82.2) | 86 (11.7)  652 (88.4) | 109 (12.0)  796 (88.0) | 147 (11.6)  1123 (88.4) | 116 (15.0)  655 (85.0) | 65 (8.8)  676 (91.2) | 89 (12.2)  638 (87.8) | 43 (7.1)  567 (93.0) | 102 (15.1)  572 (84.9) |
| **Facility level**  Dispensary  Health centre  Hospital | 604 (48.7)  411 (33.1)  226 (18.2) | 300 (34.8)  354 (41.1)  208 (24.1) | 227 (35.8)  220 (34.7)  187 (29.5) | 365 (49.5)  170 (23.0)  203 (27.5) | 451 (49.8)  283 (31.3)  171 (18.9) | 721 (56.8)  361 (28.4)  188 (14.8) | 362 (47.0)  257 (33.3)  152 (19.7) | 435 (58.7)  181 (24.4)  125(16.9) | 347 (47.7)  268 (36.9)  112 (15.4) | 354 (58.0)  160 (26.3)  96 (15.7) | 427 (63.4)  160 (23.7)  87 (12.9) |
| **Caseload on the survey day**  ≤25 patients  >25 patients | 881 (71.0)  360 (29.0) | 599 (69.5)  263 (30.5) | 554 (87.4)  80 (12.6) | 710 (96.2)  28 (3.8) | 877 (96.9)  28 (3.1) | 1148 (90.4)  122 (9.6) | 771 (100)  0 | 687 (92.7)  54 (7.3) | 670 (92.2)  57 (7.8) | 582 (95.4)  28 (4.6) | 643 (95.4)  31 (4.6) |
| **Type of malaria diagnostic at the facility**  RDT  Microscopy  Both | 120 (9.7)  1078 (86.9)  43 (3.5) | 39 (4.5)  761 (88.3)  62 (7.2) | 36 (5.7)  508 (80.1)  90 (14.2) | 101 (13.7)  614 (80.1)  23 (3.1) | 161 (17.8)  621 (68.6)  123 (13.6) | 567 (44.7)  211 (16.6)  492 (38.7) | 231 (30.0)  462 (59.9)  78 (10.1) | 353 (47.6)  183 (24.7)  205 (27.7) | 373 (51.3)  82 (11.3)  272 (37.4) | 249 (40.8)  165 (27.1)  196 (32.1) | 340 (50.5)  143 (21.2)  191 (28.3) |
| **Retrospective RDT stockouts**  No  Yes  Missing | 66 (5.3)  1175 (94.7) | 97 (11.3)  765 (88.8) | 105 (16.6)  529 (83.4) | 204 (27.6)  474 (64.2)  60 (8.1) | 205 (22.7)  699 (77.2)  1 (0.11) | 787 (62.0)  370 (29.1)  113 (8.7) | 257 (33.3)  509 (66.0)  5 (0.7) | 539 (72.7)  202 (27.3) | 537 (73.9)  183 (25.2)  7 (1.0) | 442 (72.5)  158 (25.9)  10 (1.6) | 460 (68.3)  196 (29.1)  18 (2.7) |
| **Retrospective microscopy stockouts**  No  Yes | 1031 (83.1)  210 (16.9) | 800 (92.8)  62 (7.2) | 557 (87.9)  77 (12.2) | 641 (86.9)  97 (13.1) | 821 (90.7)  84 (9.3) | 936 (73.7)  334 (26.3) | 585 (75.9)  186 (24.1) | 505 (68.2)  236 (31.9) | 575 (79.1)  152 (20.9) | 484 (79.3)  126 (20.7) | 504 (74.8)  170 (25.2) |
| **Retrospective RDT and microscopy stockouts**  No  Yes | 1071 (86.3)  170 (13.7) | 822 (95.4)  40 (4.6) | 570 (89.9)  64 (10.1) | 712 (96.5)  26 (3.5) | 859 (94.9)  46 (5.1) | 1225 (96.5)  45 (3.5) | 727 (94.3)  44 (5.7) | 728 (98.3)  13 (1.8) | 709 (97.5)  18 (2.5) | 599 (98.2)  11 (1.8) | 608 (90.2)  66 (9.8) |
| **Retrospective AL stockouts**  No  Yes  Missing | 362 (29.2)  879 (70.8) | 291 (33.8)  571 (66.2) | 309 (48.7)  325 (51.3) | 467 (63.3)  267 (36.2)  4 (0.5) | 556 (61.4)  349 (38.6) | 979 (77.1)  266 (20.9)  25 (2.0) | 398 (51.6)  368 (47.7)  5 (0.7) | 319 (43.1)  422 (57.0) | 212 (29.2)  515 (70.8) | 323 (53.0)  278 (45.6)  9 (1.5) | 307 (45.6)  353 (52.2)  15 (2.2) |
| **Malaria guidelines available**  No  Yes  Missing | 1241 (100)  0 | 833 (96.6)  29 (3.4) | 321 (50.6)  313 (49.4) | 360 (48.8)  372 (50.4)  6 (0.8) | 297 (32.8)  597 (66.0)  11 (2.2) | 539 (42.4)  731 (57.6) | 364 (47.2)  382 (49.6)  25 (3.2) | 262 (35.4)  479 (64.6) | 275 (37.8)  434 (59.7)  18 (2.5) | 137 (22.5)  473 (77.5) | 173 (25.7)  497 (73.7)  4 (0.6) |
| **Malaria new chart**  No  Yes  Missing | 1241 (100)  0 | 862 (100)  0 | 519 (81.9)  115 (18.2) | 615 (83.3)  123 (17.7) | 773 (85.4)  132 (14.6) | 982 (77.3)  288 (22.7) | 587 (76.1)  123 (16.0)  61 (7.9) | 561 (75.7)  180 (24.3) | 603 (82.9)  116 (16.0)  8 (1.1) | 404 (66.2)  194 (31.8)  12 (2.0) | 433 (64.2)  229 (34.0)  12 (1.8) |
| **Cadre dispensing drugs**  Community health workers  Nurse/ Clinician  Pharmacists /pharm techs  Others  Missing | 267 (21.5)  433 (34.9)  292 (23.5)  249 (20.1) | 152 (17.6)  331 (38.4)  283 (32.8)  96 (11.1) | 68 (10.7)  251 (39.6)  228 (36.0)  87 (13.7) | 106 (14.4)  304 (41.2)  209 (28.3)  119 (16.1) | 167 (18.5)  412 (45.5)  189 (20.9)  137 (15.1) | 194 (15.3)  545 (42.9)  262 (20.6)  269 (21.2) | 80 (10.4)  427 (55.4)  179 (23.2)  81 (10.5)  4 (0.5) | 151 (20.4)  330 (44.5)  186 (25.1)  74 (10.0) | 115 (15.8)  330 (45.4)  189 (26.0)  93 (12.8) | 31 (5.1)  252 (41.3)  189 (31.0)  123 (20.2)  15 (2.5) | 116 (17.2)  331 (49.1)  171 (25.4)  56 (8.3) |
| **Health worker level** | | | | | | | | | | | |
| **Age**, median (IQR) | 34 (28-44) | 31 (27-40) | 32 (26-43) | 33 (28-42) | 30 (27-37) | 34 (29-45) | 32 (28-40) | 31 (29-40) | 34 (30-40) | 34 (28-38) | 30 (27-34) |
| **Gender**  Male  Female | 701 (56.5)  540 (43.5) | 500 (58.0)  362 (42.0) | 314 (49.5)  320 (50.5) | 348 (47.2)  390 (52.9) | 462 (51.1)  443 (49.0) | 566 (44.6)  704 (55.4) | 453 (58.8)  318 (41.3) | 342 (46.2)  399 (53.9) | 352 (48.4)  375 (51.6) | 330 (54.1)  280 (45.9) | 352 (52.2)  322 (47.8) |
| **Facility in charge**  No  Yes  Missing | 757 (61.0)  484 (39.0) | 577 (66.9)  285 (33.1) | 398 (62.8)  236 (37.2) | 463 (62.7)  263 (35.6)  12 1.6) | 577 (63.8)  326 (36.0)  2 (0.2) | 699 (55.0)  571 (45.0) | 475 (61.6)  296 (38.4) | 455 (61.4)  286 (38.6) | 475 (65.3)  252 (34.7) | 339 (55.6)  260 (42.6)  11 (1.8) | 510 (75.7)  149 (22.1)  15 (2.2) |
| **Cadre**  Others  Nurse  Clinical / Medical officer | 0  710 (57.2)  531 (42.8) | 40 (4.6)  412 (47.8)  410 (47.6) | 8 (1.3)  361 (56.9)  265 (41.8) | 27 (3.7)  382 (51.8)  329 (44.6) | 48 (5.3)  520 (57.5)  337 (37.2) | 18 (1.4)  891 (70.2)  361 (28.4) | 41 (5.3)  391 (50.7)  339 (44.0) | 52 (7.0)  423 (57.1)  266 (35.9) | 51 (7.0)  372 (51.2)  304 (41.8) | 28 (4.6)  338 (55.4)  244 (40.0) | 51 (7.6)  310 (46.0)  313 (46.4) |
| **HW perception of endemicity**  Low  High  Missing | 209 (16.8)  1032 (83.2) | 314 (36.4)  548 (63.6) | 289 (45.6)  345 (54.4) | 295 (40.0)  415 (56.2)  28 (3.8) | 392 (43.3)  510 (56.4)  3 (0.3) | 610 (48.0)  660 (52.0) | 455 (59.0)  316 (41.0) | 356 (48.0)  385 (52.0) | 363 (49.9)  360 (49.5)  4 (0/6) | 268 (43.9)  342 (56.1) | 315 (46.7)  359 (53.3) |
| **MCM in-service training**  No  Yes | 1241 (100)  0 | 625 (72.5)  237 (27.5) | 414 (65.3)  220 (34.7) | 472 (64.0)  266 (36.0) | 634 (70.1)  271 (29.9) | 623 (49.1)  647 (50.9) | 484 (62.8)  287 (37.2) | 339 (45.8)  402 (54.3) | 350 (48.1)  377 (51.9) | 174 (28.5)  436 (71.5) | 194 (28.8)  480 (71.2) |
| **Access to current malaria diagnosis and treatment** **guidelines**  No  Yes  Missing | 1241 (100)  0 | 825 (95.7)  37 (4.3) | 346 (54.6)  288 (45.4) | 344 (46.6)  389 (52.7)  5 (0.7) | 326 (36.0)  568 (62.8)  11 (1.2) | 590 (46.5)  680 (53.5) | 362 (47.0)  385 (49.9)  24 (3.1) | 282 (38.1)  459 (61.9) | 306 (42.1)  412 (56.7)  9 (1.2) | 138 (22.6)  465 (76.2)  7 (1.2) | 203 (30.1)  467 (69.3)  4 (0.6) |
| **Access to IMCI guidelines**  No  Yes  Missing | 648 (52.2)  593 (47.8) | 396 (45.9)  466 (54.1) | 374 (59.0)  260 (41.0) | 354 (48.0)  379 (51.4)  5 (0.7) | 442 (48.8)  459 (50.7)  4 (0.4) | 552 (43.5)  716 (56.4)  2 (0.2) | 212 (27.5)  557 (72.2)  2 (0.3) | 274 (37.0)  467 (63.0) | 243 (33.4)  483 (66.4)  1 (0.1) | 132 (21.6)  478 (78.4) | 169 (25.1)  501 (74.3)  4 (0.6) |
| **Any supervision in the previous 3 months**  No  Yes | 628 (50.6)  613 (49.4) | 424 (49.2)  438 (50.8) | 203 (32.0)  431 (68.0) | 298 (40.4)  440 (59.6) | 291 (32.2)  614 (67.9) | 321 (25.3)  949 (74.7) | 370 (48.0)  401 (42.0) | 247 (33.3)  494 (66.7) | 171 (23.5)  556 (76.5) | 245 (40.2)  365 (59.8) | 203 (30.1)  471 (69.9) |
| **MCM supervision in the previous 3 months**  No  Yes | 971 (78.2)  270 (21.8) | 674 (78.2)  188 (21.8) | 410 (64.7)  224 (35.3) | 557 (75.5)  181 (24.5) | 472 (52.2)  433 (47.9) | 849 (66.9)  421 (33.2) | 653 (84.7)  118 (15.3) | 454 (61.3)  287 (38.7) | 425 (58.5)  302 (41.5) | 405 (66.4)  205 (33.6) | 287 (45.6)  387 (57.4) |
| **Observation of consultations in the previous 3 months**  No  Yes | 1163 (93.7)  78 (6.3) | 740 (85.9)  122 (14.2) | 563 (88.8)  71 (11.2) | 652 (88.4)  86 (11.7) | 718 (79.3)  187 (20.7) | 1133 (89.3)  137 (10.8) | 697 (90.4)  74 (9.6) | 591 (79.8)  150 (20.2) | 606 (83.4)  121 (16.6) | 451 (73.9)  159 (26.1) | 396 (58.8)  278 (41.3) |
| **Feedback in the previous 3 months**  No  Yes | 1129 (91.0)  112 (9.0) | 753 (87.4)  109 (12.7) | 448 (70.7)  186 (29.3) | 598 (81.0)  140 (19.0) | 551 (60.9)  354 (39.1) | 968 (76.2)  302 (23.8) | 681 (88.3)  90 (11.7) | 521 (70.3)  220 (29.7) | 488 (67.1)  239 (32.9) | 441 (72.3)  169 (27.7) | 392 (58.2)  282 (41.8) |
| **Correct knowledge on testing**  No  Yes  Missing | 0 | 114 (13.2)  748 (86.8) | 138 (21.8)  492 (77.6)  4(0.6) | 88 (11.9)  638 (86.5)  12 (1.6) | 151 (16.7)  754 (83.3) | 248 (19.5)  1015 (79.9)  7 (0.6) | 116 (15.1)  655 (85.0) | 56 (7.6)  685 (92.4) | 78 (10.7)  649 (89.3) | 80 (13.1)  530 (86.9) | 54 (8.0)  620 (92.0) |
| **Correct knowledge on malaria treatment policy**  No  Yes | 0 | 460 (53.4)  402 (46.6) | 292 (46.1)  342 (53.9) | 316 (42.8)  422 (57.2) | 256 (28.3)  649 (71.7) | 271 (21.3)  999 (78.7) | 181 (23.5)  590 (76.5) | 71 (9.6)  670 (90.4) | 77 (10.6)  650 (89.4) | 73 (12.0)  537 (88.0) | 35 (5.2)  639 (94.8) |
| **Patient-level** | | | | | | | | | | | |
| **Age**, median (IQR) | 5 (1.8-21) | 5 (1.7-27) | 5 (2-26) | 6 (2-26) | 7 (2-26) | 6 (2.5-19) | 6 (2.1-23) | 8 (2.7-22) | 6 (2-18) | 6 (1.7-23) | 8 (3-20) |
| <5 years  ≥5 years  Missing | 593 (47.8)  648 (42.2) | 420 (48.7)  442 (51.3) | 301 (47.5)  333 (52.5) | 327 (44.3)  411 (55.7) | 375 (41.4)  530 (58.6) | 536 (42.2)  733 (57.7)  1 (0.1) | 349 (45.3)  422 (54.7) | 284 (38.3)  457 (61.7) | 318 (43.7)  409 (56.3) | 279 (45.7)  331 (54.3) | 234 (34.7)  440 (65.3) |
| 0-11 months  12-59 months  5-14 years  ≥15 years  Missing | 166 (13.4)  427 (34.4)  244 (19.7)  404 (32.6) | 132 (15.3)  288 (33.4)  140 (16.2)  302 (35.0) | 63 (9.9)  238 (37.5)  109 (17.2)  224 (35.3) | 96 (13.0)  231 (31.3)  136 (18.4)  275 (37.3) | 108 (11.9)  267 (29.5)  176 (19.5)  354 (39.1) | 121 (9.5)  415 (32.7)  375 (29.5)  358 (28.2)  1 (0.1) | 94 (12.2)  255 (33.1)  180 (23.4)  242 (31.4) | 57 (7.7)  227 (30.6)  207 (27.9)  250 (33.7) | 89 (12.2)  229 (31.5)  191 (26.3)  218 (30.0) | 84 (13.8)  195 (32.0)  130 (21.3)  201 (33.0) | 40 (5.9)  194 (28.8)  214 (31.8)  226 (33.5) |
| **Duration of illness,** median (IQR) | 3 (2-4) | 3 (2-4) | 3 (2-4) | 3 (2-4) | 3 (2-4) | 3 (2-4) | 3 (2-4) | 3 (2-4) | 3 (2-4) | 3 (2-4) | 3 (2-3) |
| **Temperature**  <37.5°C  ≥37.5°C  Missing | 870 (70.1)  369 (29.7)  2 (0.2) | 595 (69.0)  266 (30.9)  1 (0.1) | 415 (65.5)  213 (33.6)  6 (1.0) | 575 (77.9)  161 (21.8)  2 (0.3) | 656 (77.5)  246 (27.2)  3 (0.3) | 822 (64.7)  447 (35.2)  1 (0.1) | 559 (72.5)  208 (27.0)  4 (0.5) | 566 (76.4)  174 (23.5)  1 (0.1) | 473 (65.1)  253 (34.8)  1 (0.1) | 410 (67.2)  197 (32.3)  3 (0.5) | 448 (66.5)  217 (32.2)  9 (1.3) |
| **Prior use of antimalarial**  No  Yes | 1165 (93.9)  76 (6.1) | 824 (95.6)  38 (4.4) | 599 (94.5)  35 (5.5) | 713 (96.6)  25 (3.4) | 851 (94.0)  54 (6.0) | 1220 (96.1)  50 (3.9) | 756 (98.1)  15 (2.0) | 722 (97.4)  9 (2.6) | 692 (95.2)  35 (4.8) | 598 (98.0)  12 (2.0) | 651 (96.6)  23 (3.4) |
| **Main complaints**  **Fever**  No  Yes  **Cough**  No  Yes  **Diarrhoea**  No  Yes  **Headache**  No  Yes  **Running** **nose**  No  Yes  **Rash**  No  Yes  **Vomiting**  No  Yes  **Chills**  No  Yes | 208 (16.7)  1033 (83.2)  692 (55.8)  549 (44.2)  1081 (87.1)  160 (12.9)  801 (64.5)  440 (35.5)  1058 (85.3)  183 (14.8)  1211 (97.6)  30 (2.4)  1049 (84.5)  192 (15.5)  1217 (98.1)  24 (1.9) | 121 (14.0)  741 (86.0)  499 (57.9)  363 (42.1)  748 (86.8)  114 (13.2)  615 (71.4)  247 (28.7)  779 (90.4)  83 (9.6)  830 (96.3)  32 (3.7)  697 (80.9)  165 (19.1)  816 (94.7)  46 (5.3) | 108 (17.0)  526 (83.0)  349 (55.1)  285 (45.0)  567 (89.4)  67 (10.6)  418 (65.9)  216 (34.1)  572 (90.2)  62 (9.8)  614 (96.9)  20 (3.2)  525 (82.8)  109 (17.2)  598 (94.3)  36 (5.7) | 114 (15.5)  624 (84.6)  412 (55.8)  326 (44.2)  642 (87.0)  96 (13.0)  489 (66.3)  249 (33.7)  661 (89.6)  77 (10.4)  725 (98.2)  13 (1.8)  618 (83.7)  120 (16.3)  704 (95.4)  34 (4.6) | 190 (21.0)  715 (79.0)  486 (53.7)  419 (46.3)  820 (90.6)  85 (9.4)  608 (67.2)  297 (32.8)  775 (85.6)  130 (14.4)  887 (98.0)  18 (2.0)  776 (85.8)  129 (14.3)  868 (95.9)  37 (4.1) | 199 (15.7)  1071 (84.3)  624 (49.1)  646 (50.9)  1149 (90.5)  121 (9.5)  804 (63.3)  466 (36.7)  1100 (86.6)  170 (13.4)  1242 (97.8)  28 (2.2)  1021 (80.4)  249 (19.6)  1184 (93.2)  86 (6.8) | 96 (12.5)  675 (87.6)  430 (55.8)  341 (44.2)  679 (88.1)  92 (11.9)  539 (69.9)  232 (30.1)  693 (89.9)  78 (10.1)  752 (97.5)  19 (2.5)  639 (82.9)  132 (17.1)  727 (94.3)  44 (5.7) | 128 (17.3)  613 (82.7)  403 (54.4)  338 (45.6)  664 (89.6)  77 (10.4)  416 (56.1)  325 (43.9)  679 (91.6)  62 (8.4)  725 (97.8)  16 (2.2)  601 (81.1)  140 (18.9)  687 (92.7)  54 (7.3) | 75 (10.3)  652 (89.7)  430 (59.2)  297 (40.9)  633 (87.1)  94 (12.9)  447 (61.5)  280 (38.5)  634 (87.2)  93 (12.8)  709 (97.5)  18 (2.5)  591 (81.3)  136 (18.7)  665 (91.5)  62 (8.5) | 55 (9.0)  555 (91.0)  317 (52.0)  293 (48.0)  538 (88.2)  72 (11.8)  383 (62.8)  227 (37.2)  521 (85.4)  89 (14.6)  591 (96.9)  19 (3.1)  499 (81.8)  111 (18.2)  576 (94.4)  34 (5.6) | 95 (14.1)  579 (85.9)  371 (55.0)  303 (45.0)  622 (92.3)  52 (7.7)  371 (55.0)  303 (45.0)  581 (86.2)  93 (13.8)  662 (98.2)  12 (1.8)  572 (84.9)  102 (15.1)  605 (89.8)  69 (10.2) |
| **Case complexity**  No fever  Fever only  Fever & other complaints | 208 (16.8)  171 (13.8)  862 (69.5) | 121 (14.0)  127 (14.7)  614 (71.2) | 108 (17.0)  87 (13.7)  439 (69.2) | 114 (15.5)  101 (13.7)  523 (70.9) | 190 (21.0)  127 (14.0)  588 (65.0) | 199 (15.7)  123 (9.7)  948 (74.7) | 96 (12.5)  121 (15.7)  554 (71.9) | 128 (17.3)  78 (10.5)  535 (72.2) | 75 (10.3)  100 (13.8)  552 (75.9) | 55 (9.0)  61 (10.0)  494 (81.0) | 95 (14.1)  76 (11.3)  503 (74.6) |
