# Supplementary material for: Determinants of improvement trends in health workers’ compliance with outpatient malaria case-management guidelines at health facilities with available “test and treat” commodities in Kenya
Source: PLoS One. 2021 Nov 5;16(11):e0259020. doi: 10.1371/journal.pone.0259020 (PMC8570506; doi:10.1371/journal.pone.0259020)
Supplement: S2 Table — *1-main effects estimate adjusting for time; 2- T-OR = unadjusted odds ratio from the covariate and time interaction; FBO/NGO- Faith-based organisation/Non-Governmental organisation; RDT-rapid diagnostics tests; AL-artemether-lumefantrine; IQR-interquartile range; HW-health worker; MCM-malaria case-management. (DOCX) [file pone.0259020.s005.docx]

|  | **Factor** | **OR (95% CI)^1^** | **P-value** | **T-OR (95% CI)^2^** | **P-value for interaction** |
| --- | --- | --- | --- | --- | --- |
| **Malaria endemicity** | **Epidemiological zone**  Lake endemic  Coast endemic  Highland epidemic  Semi-arid seasonal  Low risk | Ref  0.56 (0.36 - 0.85)  0.39 (0.28 - 0.54)  0.35 (0.26 - 0.48)  0.23 (0.17 - 0.32) | 0.007  <0.001  <0.001  <0.001 | Ref  0.69 (0.57 - 0.84)  0.82 (0.70 - 0.96)  0.55 (0.47 - 0.64)  0.54 (0.46 - 0.64) | **<0.001** |
| **Health Facility level** | **Facility ownership**  FBO/NGO  Government | Ref  0.43 (0.32 - 0.57) | **<0.001** | Ref  0.98 (0.85 - 1.13) | 0.777 |
|  | **Facility level**  Dispensary  Health centre  Hospital | Ref  1.00 (0.77 - 1.29)  0.79 (0.58 - 1.09) | 0.999  0.146 | Ref  0.99 (0.87 - 1.13)  1.09 (0.92 - 1.28) | 0.551 |
|  | **Caseload on the survey day**  ≤25 patients  >25 patients | Ref  1.11 (0.61 - 2.01) | 0.740 | Ref  1.72 (1.29 - 2.30) | **<0.001** |
|  | **Type of malaria diagnostic at the facility**  RDT  Microscopy  Both | Ref  2.37 (1.77 - 3.16)  2.69 (1.97 - 3.67) | <0.001  <0.001 | Ref  0.69 (0.58 - 0.81)  0.64 (0.52 - 0.78) | **<0.001** |
|  | **Retrospective RDT stockouts**  No  Yes | Ref  1.52 (1.17 - 1.97) | **0.002** | Ref  0.97 (0.84 - 1.11) | 0.626 |
|  | **Retrospective microscopy stockouts**  No  Yes | Ref  0.48 (0.36 - 0.63) | <0.001 | Ref  1.21 (1.04 - 1.41) | **0.013** |
|  | **Retrospective RDT and microscopy stockouts**  No  Yes | Ref  0.78 (0.48 - 1.28) | 0.330 | Ref  1.30 (1.03 - 1.63) | **0.026** |
|  | **Retrospective AL stockouts**  No  Yes | Ref  0.84 (0.67 - 1.05) | 0.119 | Ref  0.90 (0.80 - 1.01) | 0.062 |
|  | **Malaria guidelines available**  No  Yes | Ref  1.30 (1.02 - 1.66) | **0.036** | Ref  0.98 (0.86 - 1.11) | 0.742 |
|  | **Malaria new chart**  No  Yes | Ref  1.06 (0.80 - 1.41) | 0.691 | Ref  0.94 (0.79 - 1.12) | 0.509 |
| **Health worker level** | **Age, median (IQR)** | 1.00 (0.99 - 1.01) | 0.656 | 1.00 (0.99 - 1.00) | 0.849 |
|  | **Gender**  Female  Male | Ref  0.87 (0.73 - 1.03) | 0.110 | Ref  0.96 (0.87 - 1.05) | 0.341 |
|  | **Facility in charge**  No  Yes | Ref  1.15 (0.95 - 1.39) | 0.143 | Ref  0.97 (0.89 - 1.07) | 0.583 |
|  | **Cadre**  Others  Nurse  Clinical officer/ Medical officer | Ref  1.68 (1.05 - 2.68)  1.77 (1.09 - 2.85) | **0.031**  **0.020** | Ref  1.10 (0.83 - 1.46)  1.00 (0.76 - 1.33) | 0.191  0.979 |
|  | **HW perception of endemicity**  Low  High | Ref  2.22 (1.81 - 2.72) | <0.001 | Ref  1.38 (1.25 - 1.53) | **<0.001** |
|  | **MCM in-service training**  No  Yes | Ref  1.27 (1.06 - 1.54) | 0.012 | Ref  1.12 (1.01 - 1.24) | **0.037** |
|  | **Access to current malaria diagnosis and treatment guidelines**  No  Yes | Ref  1.26 (1.01 - 1.56) | **0.043** | Ref  0.99 (0.88 - 1.12) | 0.889 |
|  | **Any supervision in the previous 3 months**  No  Yes | Ref  1.13 (0.92 - 1.39) | 0.247 | Ref  1.02 (0.92 - 1.13) | 0.719 |
|  | **MCM supervision in the previous 3 months**  No  Yes | Ref  1.42 (1.16 - 1.75) | 0.001 | Ref  1.18 (1.06 - 1.31) | **0.002** |
|  | **Observation of consultations in the previous 3 months**  No  Yes | Ref  1.52 (1.15 - 2.00) | 0.003 | Ref  1.22 (1.06 - 1.40) | **0.005** |
|  | **Feedback in the previous 3 months**  No  Yes | Ref  1.77 (1.40 - 2.23) | 0.000 | Ref  1.18 (1.04 - 1.33) | **0.011** |
|  | **Correct knowledge on testing**  No  Yes | Ref  1.57 (1.21 - 2.03) | **0.001** | Ref  1.00 (0.86 - 1.17) | 0.975 |
|  | **Correct knowledge on malaria treatment policy**  No  Yes | Ref  1.48 (1.20 - 1.83) | **0.000** | Ref  0.98 (0.88 - 1.10) | 0.727 |
| **Patient-level** | | | | | |
|  | <5 years  ≥5 years | Ref  1.59 (1.43 - 1.78) | <0.001 | Ref  0.94 (0.89 - 0.99) | **0.028** |
|  | 0-11 months  12-59 months  5-14 years  ≥15 years | Ref  1.61 (1.33 - 1.94)  2.14 (1.75 - 2.62)  2.39 (1.97 - 2.89) | <0.001  <0.001  <0.001 | Ref  1.07 (0.97 - 1.18)  1.04 (0.93 - 1.15)  0.96 (0.87 - 1.06) | **0.021** |
|  | **Temperature**  <37.5°C  ≥37.5°C | Ref  1.33 (1.18 - 1.50) | <0.001 | Ref  1.11 (1.05 - 1.19) | **<0.001** |
|  | **Prior use of antimalarial**  No  Yes | Ref  1.10 (0.84 - 1.44) | 0.495 | Ref  1.06 (0.92 - 1.22) | 0.416 |
|  | **Main complaints** |  |  |  |  |
|  | **Fever**  No  Yes | Ref  1.01 (0.86 - 1.18) | 0.904 | Ref  1.08 (1.00 - 1.17) | 0.064 |
|  | **Cough**  No  Yes | Ref  0.66 (0.59 - 0.73) | **<0.001** | Ref  0.97 (0.92 - 1.03) | 0.333 |
|  | **Diarrhoea**  No  Yes | Ref  1.15 (0.97 - 1.36) | 0.115 | Ref  1.07 (0.98 - 1.17) | 0.128 |
|  | **Headache**  No  Yes | Ref  1.90 (1.69 - 2.13) | **<0.001** | Ref  1.00 (0.94 - 1.05) | 0.893 |
|  | **Running nose**  No  Yes | Ref  0.57 (0.48 - 0.67) | **0.000** | Ref  1.01 (0.93 - 1.10) | 0.812 |
|  | **Rash**  No  Yes | Ref  0.40 (0.28 - 0.58) | **<0.001** | Ref  0.93 (0.77 - 1.12) | 0.436 |
|  | **Vomiting**  No  Yes | Ref  1.33 (1.15 - 1.53) | <0.001 | Ref  1.10 (1.02 - 1.18) | **0.013** |
|  | **Chills**  No  Yes | Ref  1.62 (1.27 - 2.07) | **<0.001** | Ref  1.14 (1.00 - 1.31) | 0.054 |
|  | **Case complexity**  No fever  Fever only  Fever and other complaints | Ref  0.88 (0.71 - 1.08)  1.04 (0.89 - 1.22) | 0.209  0.650 | Ref  1.11 (1.00 - 1.24)  1.07 (0.99 - 1.16) | 0.126 |
