# Supplementary material for: Determinants of improvement trends in health workers’ compliance with outpatient malaria case-management guidelines at health facilities with available “test and treat” commodities in Kenya
Source: PLoS One. 2021 Nov 5;16(11):e0259020. doi: 10.1371/journal.pone.0259020 (PMC8570506; doi:10.1371/journal.pone.0259020)
Supplement: S3 Table — *1-main effects estimate adjusting for time; 2- T-OR = unadjusted odds ratio from the covariate and time interaction; FBO/NGO- Faith-based organisation/Non-Governmental organisation; RDT-rapid diagnostics tests; AL-artemether-lumefantrine; IQR-interquartile range; HW-health worker; MCM-malaria case-management. (DOCX) [file pone.0259020.s006.docx]

|  | **Factor** | **OR (95% CI)^1^** | **P-value** | **T-OR (95% CI)^2^** | **P-value for interaction** |
| --- | --- | --- | --- | --- | --- |
| **Malaria endemicity** | **Epidemiological zone**  Lake endemic  Coast endemic  Highland epidemic  Semi-arid seasonal  Low risk | Ref  0.25 (0.16 - 0.40)  0.27 (0.17 - 0.43)  0.18 (0.12 - 0.29)  0.11 (0.07 - 0.17) | Ref  **<0.001**  **<0.001**  **<0.001**  **<0.001** | Ref  0.66 (0.53 - 0.82)  0.78 (0.63 - 0.96)  0.55 (0.44 - 0.68)  0.52 (0.42 - 0.65) | **<0.001** |
| **Health Facility level** | **Facility ownership**  FBO/NGO  Government | Ref  0.24 (0.18 - 0.34) | **<0.001** | Ref  1.03 (0.88 - 1.20) | 0.780 |
|  | **Facility level**  Dispensary  Health centre  Hospital | Ref  1.04 (0.78 - 1.38)  0.88 (0.66 - 1.16) | 0.783  0.464 | Ref  0.96 (0.83 - 1.11)  1.02 (0.88 - 1.18) | 0.759 |
|  | **Caseload on the survey day**  ≤25 patients  >25 patients | Ref  1.07 (0.55 - 2.06) | 0.847 | Ref  1.78 (1.29 - 2.44) | **0.001** |
|  | **Type of malaria diagnostic at the facility**  RDT  Microscopy  Both | Ref  2.87 (2.09 - 3.93)  2.89 (2.11 - 3.96) | **<0.001**  **<0.001** | Ref  0.74 (0.63 - 0.88)  0.66 (0.56 - 0.78) | **<0.001** |
|  | **Retrospective RDT stockouts**  No  Yes | Ref  1.81 (1.36 - 2.41) | **<0.001** | Ref  0.97 (0.84 - 1.13) | 0.729 |
|  | **Retrospective microscopy stockouts**  No  Yes | Ref  0.41 (0.30 - 0.55) | **<0.001** | Ref  1.20 (1.03 - 1.41) | **0.023** |
|  | **Retrospective RDT and microscopy stockouts**  No  Yes | Ref  0.61 (0.36 - 1.03) | 0.064 | Ref  1.39 (1.08 - 1.79) | **0.010** |
|  | **Retrospective AL stockouts**  No  Yes | Ref  1.05 (0.82 - 1.34) | 0.699 | Ref  0.82 (0.72 - 0.93) | **0.002** |
|  | **Malaria guidelines available**  No  Yes | Ref  1.16 (0.88 - 1.51) | 0.294 | Ref  1.00 (0.87 - 1.15) | 0.975 |
|  | **Malaria new chart**  No  Yes | Ref  0.96 (0.70 - 1.32) | 0.802 | Ref  0.94 (0.78 - 1.14) | 0.524 |
| **Health worker level** | **Age, median (IQR)** | 1.00 (0.99 - 1.01) | 0.639 | 1.00 (1.00 - 1.01) | 0.846 |
|  | **Gender**  Female  Male | Ref  0.92 (0.76 - 1.11) | 0.365 | Ref  0.98 (0.89 - 1.08) | 0.734 |
|  | **Facility in charge**  No  Yes | Ref  1.16 (0.95 - 1.42) | 0.139 | Ref  1.02 (0.92 - 1.12) | 0.749 |
|  | **Cadre**  Others  Nurse  Clinical/ Medical officer | Ref  1.49 (0.90 - 2.47)  1.59 (0.96 - 2.65) | 0.126  0.077 | Ref  0.94 (0.70 - 1.26)  0.88 (0.65 - 1.18) | 0.420 |
|  | **HW perception of endemicity**  Low  High | Ref  3.38 (2.72 - 4.21) | **<0.001** | Ref  1.41 (1.27 - 1.58) | **<0.001** |
|  | **MCM in-service training**  No  Yes | Ref  1.09 (0.89 - 1.33) | 0.422 | Ref  1.07 (0.96 - 1.20) | 0.222 |
|  | **Access to current malaria diagnosis and treatment guidelines**  No  Yes | Ref  1.14 (0.90 - 1.46) | 0.279 | Ref  1.04 (0.91 - 1.19) | 0.564 |
|  | **Any supervision in the previous 3 months**  No  Yes | Ref  1.17 (0.94 - 1.46) | 0.167 | Ref  1.05 (0.94 - 1.17) | 0.412 |
|  | **MCM supervision in the previous 3 months**  No  Yes | Ref  1.66 (1.32 - 2.09) | **<0.001** | Ref  1.17 (1.04 - 1.30) | **0.009** |
|  | **Observation of consultations in the previous 3 months**  No  Yes | Ref  1.61 (1.20 - 2.18) | **<0.001** | Ref  1.19 (1.03 - 1.38) | **<0.001** |
|  | **Feedback in the previous 3 months**  No  Yes | Ref  1.91 (1.48 - 2.47) | **<0.001** | Ref  1.22 (1.07 - 1.39) | **0.005** |
|  | **Correct knowledge on testing**  No  Yes | Ref  1.73 (1.32 - 2.27) | **<0.001** | Ref  1.03 (0.88 - 1.21) | 0.724 |
| **Patient-level** | **Age (median, IQR)** | 1.01 (1.01 - 1.02) | **<0.001** | 1.00 (1.00 - 1.00) | **<0.001** |
|  | <5 years  ≥5 years | Ref  1.86 (1.66 - 2.08) | **<0.001** | Ref  0.92 (0.87 - 0.98) | **0.006** |
|  | 0-11 months  12-59 months  5-14 years  ≥15 years | Ref  1.84 (1.52 - 2.22)  2.79 (2.31 - 3.37)  3.04 (2.52 - 3.68) | **<0.001**  **<0.001**  **<0.001** | Ref  1.08 (0.98 - 1.18)  1.03 (0.93 - 1.13)  0.95 (0.86 - 1.04) | **0.004** |
|  | **Duration of illness (median IQR)** | 0.99 (0.97 - 1.01) | 0.494 | 1.00 (0.99 - 1.01) | 0.500 |
|  | **Temperature**  <37.5°C  ≥37.5°C | Ref  1.26 (1.20 - 1.33) | **<0.001** | Ref  1.15 (1.08 - 1.23) | **0.003** |
|  | **Prior use of antimalarial**  No  Yes | Ref  1.80 (1.34 - 2.44) | **<0.001** | Ref  0.95 (0.82 - 1.11) | 0.547 |
|  | **Main complaints** |  |  |  |  |
|  | **Fever**  No  Yes | Ref  1.08 (0.92 - 1.27) | 0.346 | Ref  1.08 (1.00 - 1.18) | 0.053 |
|  | **Cough**  No  Yes | Ref  0.52 (0.47 - 0.58) | **<0.001** | Ref  0.95 (0.90 - 1.00) | 0.056 |
|  | **Diarrhoea**  No  Yes | Ref  1.19 (1.00 - 1.42) | 0.052 | Ref  1.07 (0.98 - 1.17) | 0.119 |
|  | **Headache**  No  Yes | Ref  2.80 (2.48 - 3.17) | **<0.001** | Ref  0.99 (0.93 - 1.05) | 0.725 |
|  | **Running nose**  No  Yes | Ref  0.51 (0.42 - 0.60) | **<0.001** | Ref  0.96 (0.88 - 1.04) | 0.322 |
|  | **Rash**  No  Yes | Ref  0.31 (0.21 - 0.44) | **<0.001** | Ref  0.96 (0.80 - 1.15) | 0.660 |
|  | **Vomiting**  No  Yes | Ref  1.99 (1.71 - 2.32) | **<0.001** | Ref  1.11 (1.02 - 1.20) | **0.010** |
|  | **Chills**  No  Yes | Ref  2.72 (2.04 - 3.61) | **<0.001** | Ref  1.06 (0.91 - 1.23) | 0.449 |
|  | **Case complexity**  No fever  Fever only  Fever & other complaints | Ref  0.83 (0.67 - 1.02)  1.14 (0.92 - 1.41) | 0.077  0.123 | Ref  1.09 (0.98 - 1.22)  1.08 (0.97 - 1.20) | 0.171 |
