# Supplementary material for: Determinants of improvement trends in health workers’ compliance with outpatient malaria case-management guidelines at health facilities with available “test and treat” commodities in Kenya
Source: PLoS One. 2021 Nov 5;16(11):e0259020. doi: 10.1371/journal.pone.0259020 (PMC8570506; doi:10.1371/journal.pone.0259020)
Supplement: S4 Table — *1-main effects estimate adjusting for time; 2- T-OR = unadjusted odds ratio from the covariate and time interaction; FBO/NGO- Faith-based organisation/Non-Governmental organisation; RDT-rapid diagnostics tests; AL-artemether-lumefantrine; IQR-interquartile range; HW-health worker; MCM-malaria case-management. (DOCX) [file pone.0259020.s007.docx]

|  | **Factor** | **OR (95% CI)^1^** | **P-value** | **T-OR (95% CI)^2^** | **P-value for interaction** |
| --- | --- | --- | --- | --- | --- |
| **Malaria endemicity** | **Epidemiological zone**  Lake endemic  Coast endemic  Highland epidemic  Semi-arid seasonal  Low risk | Ref  1.97 (0.65 - 5.99)  1.20 (0.54 - 2.67)  1.95 (0.90 - 4.21)  1.90 (0.66 - 5.51) | 0.231  0.652  0.090  0.237 | Ref  1.14 (0.66 - 1.96)  0.76 (0.51 - 1.13)  0.65 (0.44 - 0.95)  0.45 (0.27 - 0.75) | **0.007** |
| **Health Facility level** | **Facility ownership**  FBO/NGO  Government | Ref  3.04 (1.65 - 5.57) | **<0.001** | Ref  1.11 (0.82 - 1.49) | 0.495 |
|  | **Facility level**  Dispensary  Health centre  Hospital | Ref  0.78 (0.42 - 1.45)  0.52 (0.25 - 1.09) | 0.424  0.084 | Ref  1.10 (0.80 - 1.50)  1.37 (0.92 - 2.04) | 0.289 |
|  | **Caseload on the survey day**  ≤25 patients  >25 patients | Ref  1.26 (0.43 - 3.69) | 0.672 | Ref  1.40 (0.77 - 2.56) | 0.254 |
|  | **Retrospective AL stockouts**  No  Yes | Ref  0.53 (0.30 - 0.94) | **0.030** | Ref  0.97 (0.72 - 1.30) | 0.834 |
|  | **Malaria guidelines available**  No  Yes | Ref  0.66 (0.35 - 1.23) | 0.189 | Ref  1.38 (0.99 - 1.92) | 0.056 |
|  | **Malaria new chart**  No  Yes | Ref  0.91 (0.42 - 1.97) | 0.816 | Ref  0.99 (0.63 - 1.58) | 0.987 |
| **Health worker level** | **Age, median (IQR)** | 0.99 (0.96 - 1.01) | 0.273 | 1.00 (0.99 - 1.01) | 0.839 |
|  | **Gender**  Female  Male | Ref  1.02 (0.62 - 1.67) | 0.940 | Ref  1.02 (0.80 - 1.32) | 0.845 |
|  | **Facility in charge**  No  Yes | Ref  1.22 (0.75 - 1.99) | 0.416 | Ref  0.90 (0.70 - 1.14) | 0.392 |
|  | **Cadre**  Others  Nurse  Clinical officer/ Medical officer | Ref  0.68 (0.13 - 3.62)  0.49 (0.09 - 2.69) | 0.653  0.413 | Ref  2.09 (0.71 - 6.13)  1.88 (0.64 - 5.53) | 0.298 |
|  | **HW perception of endemicity**  Low  High | Ref  0.70 (0.38 - 1.31) | 0.263 | Ref  1.48 (1.08 - 2.03) | **0.015** |
|  | **MCM in-service training**  No  Yes | Ref  1.61 (0.92 - 2.80) | 0.093 | Ref  1.51 (1.11 - 2.06) | **0.008** |
|  | **Access to current malaria diagnosis and treatment guidelines**  No  Yes | Ref  0.75 (0.42 - 1.35) | 0.342 | Ref  1.51 (1.08 - 2.09) | **0.014** |
|  | **Any supervision in the previous 3 months**  No  Yes | Ref  1.26 (0.72 - 2.21) | 0.421 | Ref  0.96 (0.73 - 1.28) | 0.800 |
|  | **MCM supervision in the previous 3 months**  No  Yes | Ref  0.89 (0.52 - 1.52) | 0.670 | Ref  1.28 (0.97 - 1.69) | 0.074 |
|  | **Observation of consultations in the previous 3 months**  No  Yes | Ref  1.02 (0.51 - 2.07) | 0.945 | 1.37 (0.96 - 1.94) | 0.077 |
|  | **Feedback in the previous 3 months**  No  Yes | Ref  0.90 (0.49 - 1.65) | 0.734 | Ref  1.26 (0.92 - 1.73) | 0.141 |
|  | **Correct knowledge on malaria treatment policy**  No  Yes | Ref  1.44 (0.82 - 2.55) | 0.208 | Ref  1.34 (1.00 - 1.80) | 0.053 |
| **Patient-level** | **Age (median, IQR)** | - 1. (1.00 - 1.02) | 0.026 | 1.00 (0.99 - 1.00) | 0.687 |
|  | <5 years  ≥5 years | Ref  1.44 (1.00 - 2.08) | **0.049** | Ref  1.00 (0.83 - 1.20) | 0.974 |
|  | 0-11 months  12-59 months  5-14 years  ≥15 years | Ref  1.15 (0.56 - 2.34)  1.40 (0.68 - 2.90)  1.83 (0.90 - 3.73) | 0.703  0.363  0.098 | Ref  1.04 (0.71 - 1.51)  1.06 (0.72 - 1.55)  1.03 (0.71 - 1.50) | 0.992 |
|  | **Duration of illness (median IQR)** | 1.02 (0.94 - 1.09) | 0.665 | 0.99 (0.95 - 1.02) | 0.450 |
|  | **Temperature**  <37.5°C  ≥37.5°C | Ref  0.65 (0.46 - 0.93) | **0.018** | 1.03 (0.86 - 1.24) | 0.717 |
|  | **Prior use of antimalarial**  No  Yes | Ref  0.50 (0.25 - 0.97) | 0.041 | Ref  1.26 (0.86 - 1.84) | 0.221 |
|  | **Main complaints** |  |  |  |  |
|  | **Fever**  No  Yes | Ref  0.77 (0.47 - 1.27) | 0.304 | Ref  0.88 (0.68 - 1.15) | 0.348 |
|  | **Cough**  No  Yes | Ref  0.98 (0.67 - 1.44) | 0.928 | Ref  1.12 (0.92 - 1.36) | 0.274 |
|  | **Diarrhoea**  No  Yes | Ref  0.79 (0.45 - 1.38) | 0.402 | Ref  1.08 (0.80 - 1.45) | 0.622 |
|  | **Headache**  No  Yes | Ref  1.04 (0.73 - 1.48) | 0.823 | Ref  1.10 (0.92 - 1.32) | 0.280 |
|  | **Running nose**  No  Yes | Ref  1.02 (0.50 - 2.08) | 0.959 | Ref  1.14 (0.79 - 1.65) | 0.473 |
|  | **Vomiting**  No  Yes | Ref  0.52 (0.36 - 0.76) | **0.001** | Ref  0.92 (0.75 - 1.12) | 0.394 |
|  | **Chills**  No  Yes | Ref  0.66 (0.35 - 1.24) | 0.192 | Ref  1.29 (0.92 - 1.82) | 0.138 |
|  | **Case complexity**  No fever  Fever only  Fever & other complaints | Ref  1.26 (0.62 - 2.58)  0.71 (0.43 - 1.18) | 0.527  0.184 | Ref  0.82 (0.57 - 1.18)  0.90 (0.69 - 1.18) | 0.561 |
