# Supplementary material for: Determinants of improvement trends in health workers’ compliance with outpatient malaria case-management guidelines at health facilities with available “test and treat” commodities in Kenya
Source: PLoS One. 2021 Nov 5;16(11):e0259020. doi: 10.1371/journal.pone.0259020 (PMC8570506; doi:10.1371/journal.pone.0259020)
Supplement: S5 Table — *1-main effects estimate adjusting for time; 2- T-OR = unadjusted odds ratio from the covariate and time interaction; FBO/NGO- Faith-based organisation/Non-Governmental organisation; RDT-rapid diagnostics tests; AL-artemether-lumefantrine; IQR-interquartile range; HW-health worker; MCM-malaria case-management. (DOCX) [file pone.0259020.s008.docx]

|  | **Factor** | **OR (95% CI)^1^** | **P-value** | **T-OR (95% CI)^2^** | **P-value for interaction** |
| --- | --- | --- | --- | --- | --- |
| **Malaria endemicity** | **Epidemiological zone**  Lake endemic  Coast endemic  Highland epidemic  Semi-arid seasonal  Low risk | Ref  14.28 (5.10 - 39.98)  1.12 (0.63 - 2.00)  2.05 (1.12 - 3.75)  6.69 (3.28 - 13.63) | <0.001  0.702  0.020  <0.001 | Ref  1.53 (0.83 - 2.81)  0.88 (0.65 - 1.19)  0.66 (0.48 - 0.90)  1.11 (0.75 - 1.65) | **0.010** |
| **Health Facility level** | **Facility ownership**  FBO/NGO  Government | Ref  1.10 (0.63 - 1.94) | 0.734 | Ref  1.08 (0.82 - 1.43) | 0.587 |
|  | **Facility level**  Dispensary  Health centre  Hospital | Ref  0.85 (0.51 - 1.42)  0.57 (0.31 - 1.04) | 0.525  0.067 | Ref  1.30 (0.98 - 1.72)  1.03 (0.76 - 1.41) | 0.173 |
|  | **Caseload on the survey day**  ≤25 patients  >25 patients | Ref  0.54 (0.20 - 1.49) | 0.236 | Ref  1.84 (0.97 - 3.48) | **0.048** |
|  | **Type of malaria diagnostic at the facility**  RDT  Microscopy  Both | Ref  0.97 (0.54 - 1.75)  2.46 (1.26 - 4.83) | 0.927  **0.009** | Ref  0.67 (0.47 - 0.94)  0.85 (0.55 - 1.31) | 0.052 |
|  | **Retrospective RDT stockouts**  No  Yes | Ref  0.72 (0.43 - 1.22) | 0.227 | 1.26 (0.98 - 1.62) | 0.061 |
|  | **Retrospective microscopy stockouts**  No  Yes | Ref  0.91 (0.51 - 1.64) | 0.755 | Ref  1.11 (0.80 - 1.54) | 0.542 |
|  | **Retrospective RDT and microscopy stockouts**  No  Yes | Ref  1.25 (0.44 - 3.56) | 0.674 | Ref  0.77 (0.49 - 1.21) | 0.268 |
|  | **Retrospective AL stockouts**  No  Yes | Ref  0.44 (0.28 - 0.68) | **<0.001** | Ref  1.22 (0.96 - 1.54) | 0.113 |
|  | **Malaria guidelines available**  No  Yes | Ref  1.96 (1.21 - 3.18) | **0.007** | Ref  0.94 (0.71 - 1.22) | 0.647 |
|  | **Malaria new chart**  No  Yes | Ref  1.39 (0.76 - 2.55) | 0.288 | Ref  1.02 (0.70 - 1.49) | 0.916 |
| **Health worker level** | **Age, median (IQR)** | 1.01 (0.98 - 1.03) | 0.590 | 0.99 (0.98 - 1.01) | 0.311 |
|  | **Gender**  Female  Male | Ref  0.73 (0.51 - 1.06) | 0.096 | Ref  0.80 (0.65 - 0.98) | **0.035** |
|  | **Facility in charge**  No  Yes | Ref  1.13 (0.75 - 1.70) | 0.552 | Ref  0.83 (0.67 - 1.03) | 0.096 |
|  | **Cadre**  Others  Nurse  Clinical officer/ Medical officer | Ref  2.55 (1.00 - 6.49)  2.58 (0.98 - 6.81) | 0.050  0.056 | Ref  1.60 (0.91 - 2.82)  1.50 (0.84 - 2.66) | 0.256 |
|  | **HW perception of endemicity**  Low  High | Ref  0.34 (0.22 - 0.53) | **<0.001** | Ref  1.02 (0.81 - 1.29) | 0.869 |
|  | **MCM in-service training**  No  Yes | Ref  2.26 (1.51 - 3.37) | <0.001 | Ref  1.44 (1.12 - 1.84) | **0.003** |
|  | **Access to current malaria diagnosis and treatment guidelines**  No  Yes | Ref  1.81 (1.18 - 2.79) | **0.007** | Ref  0.90 (0.70 - 1.16) | 0.427 |
|  | **Any supervision in the previous 3 months**  No  Yes | Ref  1.07 (0.70 - 1.63) | 0.749 | Ref  0.87 (0.69 - 1.10) | 0.255 |
|  | **MCM supervision in the previous 3 months**  No  Yes | Ref  0.81 (0.53 - 1.24) | 0.339 | Ref  1.09 (0.87 - 1.38) | 0.463 |
|  | **Observation of consultations in the previous 3 months**  No  Yes | Ref  1.13 (0.64 - 2.02) | 0.671 | Ref  1.11 (0.82 - 1.50) | 0.500 |
|  | **Feedback in the previous 3 months**  No  Yes | Ref  1.28 (0.78 - 2.10) | 0.324 | Ref  1.05 (0.80 - 1.39) | 0.729 |
|  | **Correct knowledge on testing**  No  Yes | Ref  4.31 (2.94 - 6.33) | <0.001 | Ref  1.40 (1.13 - 1.73) | **0.002** |
| **Patient-level** | **Age (median, IQR)** | 1.00 (1.00 - 1.01) | 0.421 | 1.00 (0.99 - 1.00) | 0.145 |
|  | <5 years  ≥5 years | Ref  0.77 (0.59 - 1.01) | 0.063 | Ref  0.87 (0.75 - 1.01) | 0.071 |
|  | 0-11 months  12-59 months  5-14 years  ≥15 years | Ref  0.80 (0.50 - 1.29)  0.52 (0.31 - 0.86)  0.75 (0.47 - 1.21) | 0.366  **0.010**  0.235 | Ref  0.95 (0.72 - 1.26)  0.92 (0.69 - 1.23)  0.80 (0.61 - 1.05) | 0.182 |
|  | **Duration of illness** (median IQR) | 1.06 (1.00 - 1.12) | 0.052 | 1.00 (0.97 - 1.03) | 0.984 |
|  | **Temperature**  <37.5°C  ≥37.5°C | Ref  0.57 (0.43 - 0.76) | **<0.001** | Ref  0.96 (0.83 - 1.13) | 0.660 |
|  | **Main complaints** |  |  |  |  |
|  | **Fever**  No  Yes | Ref  0.65 (0.44 - 0.95) | 0.024 | Ref  1.27 (1.03 - 1.56) | **0.028** |
|  | **Cough**  No  Yes | Ref  2.20 (1.68 - 2.88) | **<0.001** | Ref  1.08 (0.93 - 1.26) | 0.297 |
|  | **Diarrhoea**  No  Yes | Ref  1.16 (0.79 - 1.71) | 0.457 | Ref  1.06 (0.86 - 1.32) | 0.574 |
|  | **Headache**  No  Yes | Ref  0.53 (0.40 - 0.69) | <0.001 | Ref  0.85 (0.74 - 0.98) | **0.030** |
|  | **Running nose**  No  Yes | Ref  1.32 (0.86 - 2.04) | 0.206 | Ref  1.26 (0.98 - 1.62) | 0.061 |
|  | **Rash**  No  Yes | Ref  0.66 (0.25 - 1.73) | 0.392 | Ref  1.09 (0.62 - 1.91) | 0.753 |
|  | **Vomiting**  No  Yes | Ref  0.42 (0.30 - 0.59) | <0.001 | Ref  1.23 (1.01 - 1.49) | **0.036** |
|  | **Chills**  No  Yes | Ref  0.53 (0.32 - 0.88) | **0.014** | Ref  0.98 (0.75 - 1.30) | 0.907 |
|  | **Case complexity**  No fever  Fever only  Fever & other complaints | Ref  0.72 (0.43 - 1.20)  0.63 (0.43 - 0.93) | 0.205  0.020 | Ref  1.69 (1.25 - 2.29)  1.22 (0.98 - 1.50) | **0.002** |
