# Supplementary material for: Determinants of improvement trends in health workers’ compliance with outpatient malaria case-management guidelines at health facilities with available “test and treat” commodities in Kenya
Source: PLoS One. 2021 Nov 5;16(11):e0259020. doi: 10.1371/journal.pone.0259020 (PMC8570506; doi:10.1371/journal.pone.0259020)
Supplement: S6 Table — *1-main effects estimate adjusting for time; 2- T-OR = unadjusted odds ratio from the covariate and time interaction; FBO/NGO- Faith-based organisation/Non-Governmental organisation; RDT-rapid diagnostics tests; AL-artemether-lumefantrine; IQR-interquartile range; HW-health worker; MCM-malaria case-management. (DOCX) [file pone.0259020.s009.docx]

|  | **Factor** | **OR (95% CI)^1^** | **P-value** | **T-OR (95% CI)^2^** | **P-value for interaction** |
| --- | --- | --- | --- | --- | --- |
| **Malaria endemicity** | **Epidemiological zone**  Lake endemic  Coast endemic  Highland epidemic  Semi-arid seasonal  Low risk | Ref  12.09 (3.23 - 45.22)  0.38 (0.15 - 0.91)  0.18 (0.07 - 0.44)  0.47 (0.14 - 1.54) | <0.001  0.031  <0.001  0.213 | Ref  2.15 (1.17 - 3.97)  0.54 (0.35 - 0.83)  0.62 (0.40 - 0.96)  0.63 (0.34 - 1.16) | **0.019** |
| **Health Facility level** | **Facility ownership**  FBO/NGO  Government | Ref  0.44 (0.19 - 1.02) | 0.056 | Ref  1.63 (1.08 - 2.46) | 0.091 |
|  | **Facility level**  Dispensary  Health centre  Hospital | Ref  0.46 (0.22 - 0.97)  0.02 (0.01 - 0.05) | **0.042**  **<0.001** | Ref  1.11 (0.77 - 1.60)  0.74 (0.47 - 1.16) | 0.738 |
|  | **Caseload on the survey day**  ≤25 patients  >25 patients | Ref  0.13 (0.03 - 0.49) | **0.003** | Ref  0.97 (0.48 - 1.97) | 1.000 |
|  | **Retrospective AL stockouts**  No  Yes | Ref  0.66 (0.35 - 1.28) | 0.221 | Ref  1.36 (0.98 - 1.89) | 0.193 |
|  | **Cadre dispensing drugs**  Community health workers  Nurse/ Clinician  Pharmacists /pharm techs  Others | Ref  0.84 (0.34 - 2.07)  0.04 (0.01 - 0.13)  0.87 (0.29 - 2.66) | 0.707  <0.001  0.810 | Ref  0.57 (0.36 - 0.90)  0.46 (0.27 - 0.76)  0.71 (0.41 - 1.22) | 0.269 |
|  | **Malaria guidelines available**  No  Yes | Ref  3.42 (1.60 - 7.33) | **0.002** | Ref  1.01 (0.69 - 1.47) | 0.961 |
| **Health worker level** | **Age, median (IQR)** | 1.02 (0.99 - 1.04) | 0.280 | 0.99 (0.98 - 1.00) | 0.274 |
|  | **Gender**  Female  Male | Ref  0.50 (0.31 - 0.82) | **0.006** | Ref  0.92 (0.72 - 1.17) | 0.557 |
|  | **Facility in charge**  No  Yes | Ref  1.14 (0.70 - 1.85) | 0.601 | Ref  1.20 (0.94 - 1.52) | 0.228 |
|  | **Cadre**  Others  Nurse  Clinical officer/ Medical officer | Ref  5.52 (1.60 - 19.06)  2.36 (0.65 - 8.59) | **0.007**  0.192 | Ref  1.10 (0.52 - 2.35)  1.42 (0.66 - 3.08) | 0.254 |
|  | **HW perception of endemicity**  Low  High | Ref  1.78 (0.94 - 3.38) | 0.076 | Ref  1.48 (1.06 - 2.06) | 0.076 |
|  | **MCM in-service training**  No  Yes | Ref  1.49 (0.87 - 2.57) | 0.147 | Ref  1.17 (0.88 - 1.56) | 0.456 |
|  | **Access to current malaria diagnosis and treatment guidelines**  No  Yes | Ref  1.97 (1.00 - 3.88) | 0.051 | Ref  1.32 (0.92 - 1.92) | 0.388 |
|  | **Access to IMCI guidelines**  No  Yes | Ref  1.35 (0.78 - 2.33) | 0.278 | Ref  1.65 (1.25 - 2.19) | **0.004** |
|  | **Any supervision in the previous 3 months**  No  Yes | Ref  3.10 (1.70 - 5.64) | **<0.001** | Ref  1.22 (0.90 - 1.65) | 0.609 |
|  | **MCM supervision in the previous 3 months**  No  Yes | Ref  2.64 (1.50 - 4.66) | **0.001** | 1.06 (0.80 - 1.40) | 0.687 |
|  | **Observation of consultations in the previous 3 months**  No  Yes | Ref  3.27 (1.47 - 7.29) | **0.004** | Ref  1.16 (0.80 - 1.68) | 1.000 |
|  | **Feedback in the previous 3 months**  No  Yes | Ref  2.84 (1.45 - 5.57) | **0.002** | Ref  0.96 (0.69 - 1.34) | 0.721 |
| **Patient-level** | **Age (median, IQR)** | 0.99 (0.98-1.00) | 0.067 | 1.00 (1.00-1.00) | 0.527 |
|  | <5 years  ≥5 years | Ref  0.83 (0.62 - 1.10) | 0.199 | Ref  1.11 (0.97 - 1.27) | 0.137 |
|  | 0-11 months  12-59 months  5-14 years  ≥15 years | Ref  1.25 (0.74 - 2.14)  1.24 (0.71 - 2.14)  0.80 (0.46 - 1.38) | 0.406  0.447  0.414 | Ref  1.14 (0.86 - 1.50)  1.35 (1.02 - 1.78)  1.11 (0.83 - 1.47) | 0.062 |
|  | **Temperature**  <37.5°C  ≥37.5°C | Ref  2.23 (1.65 - 3.01) | **<0.001** | Ref   - 1. (0.88 - 1.16) | 0.971 |
|  | **Main complaints** |  |  |  |  |
|  | **Fever**  No  Yes | Ref  1.05 (0.70 - 1.57) | 0.823 | Ref  1.13 (0.93 - 1.38) | 0.261 |
|  | **Cough**  No  Yes | Ref  1.10 (0.83 - 1.47) | 0.511 | Ref  1.04 (0.91 - 1.19) | 0.594 |
|  | **Diarrhoea**  No  Yes | Ref  0.73 (0.46 - 1.15) | 0.172 | Ref  0.93 (0.75 - 1.15) | 0.511 |
|  | **Headache**  No  Yes | Ref  1.18 (0.89 - 1.56) | 0.259 | Ref  1.12 (0.98 - 1.28) | 0.098 |
|  | **Running nose**  No  Yes | Ref  1.00 (0.60 - 1.67) | 0.997 | Ref  0.89 (0.70 - 1.14) | 0.366 |
|  | **Rash**  No  Yes | Ref  1.23 (0.43 - 3.54) | 0.701 | Ref  0.79 (0.46 - 1.37) | 0.410 |
|  | **Vomiting**  No  Yes | Ref  1.03 (0.74 - 1.42) | 0.879 | Ref  1.01 (0.87 - 1.18) | 0.887 |
|  | **Chills**  No  Yes | Ref  1.28 (0.79 - 2.09) | 0.322 | Ref  1.18 (0.90 - 1.56) | 0.230 |
|  | **Case complexity**  No fever  Fever only  Fever & other complaints | Ref  1.05 (0.61 - 1.80)  1.05 (0.70 - 1.58) | 0.868  0.824 | Ref  0.85 (0.65 - 1.12)  1.18 (0.96 - 1.45) | **0.006** |
